# Post-stroke glymphatic and meningeal lymphatic dysfunction: mechanisms, imaging evidence, and translational challenges

**DOI:** 10.3389/fstro.2026.1861562

**Published:** 2026-07-31

**Authors:** Jin Yang, Ting Wang, Yaoyue Hu, Cuiying Liu, Tianliang Shi, Heng Zhao

**Affiliations:** 1 Department of Radiology, Tongren People's Hospital, Tongren, Guizhou, China; 2 Beijing Institute of Brain Disorders, Laboratory of Brain Disorders, Ministry of Science and Technology, Joint Innovation Center for Brain Disorders, Capital Medical University, Beijing, China; 3 Department of Ophthalmology, Tongren People's Hospital, Tongren, Guizhou, China; 4 Department of Anesthesiology, Tongren People's Hospital, Tongren, Guizhou, China; 5 School of Nursing, Capital Medical University, Beijing, China

**Keywords:** glymphatic system, magnetic resonance imaging, meningeal lymphatic system, neuroinflammation, stroke, waste clearance

## Abstract

Stroke may disrupt brain fluid and waste clearance pathways, including the glymphatic and meningeal lymphatic systems, across ischemic stroke, intracerebral hemorrhage, and subarachnoid hemorrhage. This review synthesizes current evidence on post-stroke alterations in these pathways, with emphasis on subtype-specific mechanisms, the distinction between experimental and human evidence, and the strengths and limitations of available imaging approaches. Experimental studies suggest that ischemic stroke may involve hyperacute perivascular cerebrospinal fluid influx followed by impaired glymphatic transport, that intracerebral hemorrhage is more closely linked to blood-product-related disruption of glymphatic and meningeal lymphatic drainage, and that subarachnoid hemorrhage may involve combined glymphatic and meningeal lymphatic failure. We further review *in vivo* imaging methods, particularly magnetic resonance imaging (MRI) approaches such as dynamic contrast-enhanced MRI, diffusion tensor image analysis along the perivascular space, and structural perivascular space imaging, and we discuss their biological directness and translational limitations. Across modalities, the most direct evidence still comes mainly from experimental tracer-based MRI and selected intrathecal contrast-enhanced MRI studies in humans. Among stroke subtypes, subarachnoid hemorrhage currently provides the most direct human MRI evidence of combined clearance-pathway dysfunction, whereas most other human stroke data rely on indirect surrogate markers, especially diffusion tensor image analysis along the perivascular space. Accordingly, current clearance-pathway imaging should be regarded primarily as a mechanistic and early translational research tool rather than a validated clinical biomarker. Importantly, most proposed links between molecular mechanisms, imaging readouts, and clinical outcomes remain inferential and require prospective validation in human stroke cohorts. Prospective human studies are needed to test whether targeting brain clearance pathways improves post-stroke recovery and cognition.

## 1 Introduction

Stroke remains a leading cause of death and long-term disability worldwide. In 2021, there were an estimated 11.9 million incident strokes and 7.3 million stroke-related deaths globally, and stroke was the third most common GBD level 3 cause of death worldwide (GBD 2021 Stroke Risk Factor Collaborators, 2024). Beyond the initial ischemic or hemorrhagic insult, stroke triggers secondary pathophysiological processes, including cerebral edema (Gu et al., 2022), blood-brain barrier (BBB) disruption, and a robust neuroinflammatory response (Huang et al., 2020; Candelario-Jalil et al., 2022; Lauzier et al., 2023; Chen et al., 2021). These processes can disturb cerebrospinal fluid (CSF) and interstitial fluid (ISF) circulation, impair waste clearance, and contribute to toxic protein accumulation, inflammation, and neuronal injury (Cai et al., 2024).

The glymphatic system and the meningeal lymphatic system are two complementary pathways that contribute to brain fluid and waste homeostasis (Louveau et al., 2017). The glymphatic system, first described in 2012 (Iliff et al., 2012), functions as a brain-wide perivascular network that facilitates CSF-ISF exchange and clears metabolic waste from the parenchyma. The meningeal lymphatic system, independently discovered in 2015 (Louveau et al., 2015; Aspelund et al., 2015), consists of lymphatic vessels in the dura mater that collect CSF and immune cells from the subarachnoid space and drain them to the deep cervical lymph nodes (dCLNs). Under physiological conditions, these systems are considered functionally linked and together contribute to fluid homeostasis in the central nervous system (CNS) (Louveau et al., 2017; Salvador et al., 2024).

However, stroke may disrupt both glymphatic and meningeal lymphatic pathways. Available evidence suggests associations with edema, neuroinflammation, delayed clearance, and selected functional or cognitive outcomes (Goulay et al., 2020; Back et al., 2020; Yang L. et al., 2024; Xia et al., 2024; Oka et al., 2026), but these associations remain incompletely validated in patients with stroke. Key questions therefore include how stroke subtype and disease stage influence these pathways, which imaging markers are sufficiently specific and interpretable, and whether interventions targeting these pathways can improve clinically relevant outcomes. This review therefore aims to summarize post-stroke changes in the glymphatic and meningeal lymphatic systems, distinguish clinical human studies, investigational human imaging evidence, and experimental animal evidence, and critically assess the strengths and limitations of current imaging approaches for mechanistic interpretation and translational relevance. Because the field remains at an early translational stage, this review does not treat glymphatic or meningeal lymphatic imaging as established clinical biomarkers. Instead, we critically distinguish experimental mechanisms, investigational human imaging evidence, and hypotheses that require further validation.

## 2 Literature search and review approach

We searched PubMed, Web of Science, and Scopus for peer-reviewed, English-language articles using combinations of keywords related to stroke [stroke, ischemic stroke, intracerebral hemorrhage (ICH), and subarachnoid hemorrhage (SAH)], brain clearance pathways [glymphatic, meningeal lymphatic, perivascular space (PVS), and cervical lymph nodes (CLNs)], imaging methods [diffusion tensor image analysis along the perivascular space (DTI-ALPS), dynamic contrast-enhanced magnetic resonance imaging (DCE-MRI), CSF flow, positron emission tomography (PET), single-photon emission computed tomography (SPECT), and optical imaging], and candidate molecular targets [vascular endothelial growth factor-C (VEGF-C) and aquaporin-4 (AQP4)]. The search covered studies published through May 2026 and was supplemented by manual screening of the reference lists of relevant reviews and primary articles.

Titles and abstracts were screened for relevance, followed by full-text assessment of potentially eligible articles. We prioritized studies that directly evaluated glymphatic or meningeal lymphatic structure or function after stroke, used imaging- or tracer-based measures of CSF/ISF transport, or linked clearance-pathway changes to edema, inflammation, cognition, or functional outcomes. We excluded non-peer-reviewed sources, unpublished data, and studies on unrelated neurological diseases unless they were cited to provide methodological context for imaging approaches. No new patient-level data were generated or analyzed for this review. Key study characteristics extracted for synthesis included species/population, stroke subtype/model, timing after injury, imaging modality, main outcome measures, and major limitations.

## 3 Structure of the glymphatic and meningeal lymphatic systems

**Figure 1:**
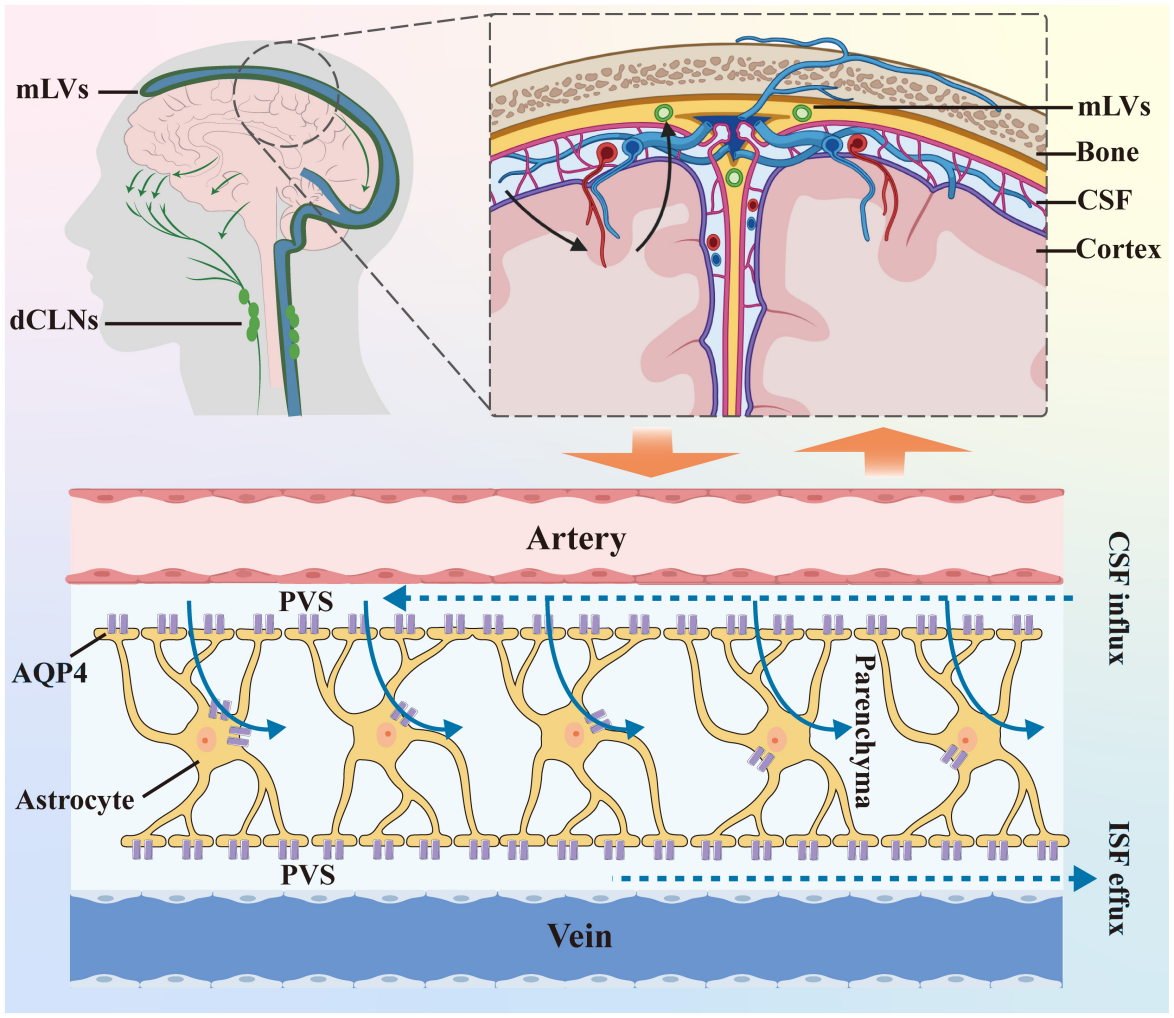
This illustration depicts the anatomical and functional organization of the glymphatic and meningeal lymphatic systems under physiological conditions. CSF enters the brain along PVS and penetrates the parenchyma via AQP4 water channels expressed on astrocytic endfeet. ISF and metabolic waste are cleared from the brain parenchyma via perivenous pathways. These waste products eventually drain into the mLVs located within the dura mater and then to the dCLNs. CSF, cerebrospinal fluid; AQP4, aquaporin-4; PVS, perivascular spaces; ISF, interstitial fluid; mLVs, meningeal lymphatic vessels; dCLNs, deep cervical lymph nodes.

Before discussing stroke-induced changes, we first outline the normal architecture of the brain's clearance pathways, including the glymphatic and meningeal lymphatic systems (Figure 1).

### 3.1 The glymphatic system

The glymphatic system is a brain-wide perivascular clearance pathway that facilitates CSF-ISF exchange and the removal of interstitial solutes (Iliff et al., 2012; Ding et al., 2023). It is often described as a lymphatic-like, rather than a true lymphatic, pathway within the CNS. Anatomically, it is organized around PVS and astrocytic endfeet (Ding et al., 2023; Wardlaw et al., 2020). PVS are fluid-filled channels surrounding penetrating vessels, particularly arterioles and venules (Wardlaw et al., 2020). Their inner boundary is formed by the vascular wall, whereas the outer boundary is formed by astrocytic endfeet and associated basement membranes (Ding et al., 2023; Wardlaw et al., 2020). At the capillary level, the anatomy becomes less distinct, and whether a continuous pericapillary compartment exists remains debated (Wardlaw et al., 2020; Ineichen et al., 2022). The vascular basement membrane is rich in laminin, collagen IV, and other extracellular matrix components (Iliff et al., 2012; Ding et al., 2023; Szczygielski et al., 2021). Astrocytic endfeet cover a large proportion of the cerebrovascular surface and form small inter-endfoot clefts that may permit fluid and solute exchange (Iliff et al., 2012; Ding et al., 2023; Szczygielski et al., 2021). At the brain surface, the glia limitans lies beneath the pia mater and contributes to the boundary between the parenchyma and surrounding CSF-containing spaces (Wardlaw et al., 2020). The water channel AQP4 is highly enriched in perivascular astrocytic endfeet and adjacent glial interfaces, supporting polarized water transport at the blood-brain and CSF-brain interfaces (Szczygielski et al., 2021; Nagelhus and Ottersen, 2013). Current models propose that arterial pulsatility contributes to periarterial CSF movement and that solute exchange occurs along perivascular or paravascular routes (Wardlaw et al., 2020; Mestre et al., 2018; Han et al., 2024). Taken together, these structures form the anatomical basis of glymphatic transport.

### 3.2 The meningeal lymphatic system

By contrast, the meningeal lymphatic system comprises conventional lymphatic vessels in the dura mater that run alongside the dural venous sinuses and meningeal arteries (Louveau et al., 2015; Aspelund et al., 2015). Their morphology varies by location, with dorsal and basal meningeal lymphatic vessels (mLVs) showing distinct structural features (Salvador et al., 2024; Cavdar et al., 2023). In particular, lymphatic vessels accompanying both dorsal and basal dural sinuses have been described in the human brain (Cavdar et al., 2023). The mLVs connect to extracranial lymphatic networks and drain toward the dCLNs (Louveau et al., 2015; Aspelund et al., 2015; Salvador et al., 2024; Eide et al., 2018). Outflow or clearance through cribriform plate lymphatics has also been described in experimental studies (Madarasz et al., 2024). Functionally, meningeal lymphatics are considered an important outflow pathway for CSF, solutes, and immune cells leaving the CNS (Eide et al., 2018). In this framework, glymphatic transport is thought to deliver fluid and waste toward the brain's borders (Iliff et al., 2012; Yankova et al., 2021), after which meningeal lymphatics participate in their clearance into peripheral lymphatic pathways (Louveau et al., 2015; Aspelund et al., 2015; Yankova et al., 2021). The identification of meningeal lymphatics challenged the traditional concept of a CNS entirely lacking lymphatic drainage and provided a structural basis for brain-immune communication.

## 4 Stroke-induced alterations in brain clearance pathways

**Figure 2:**
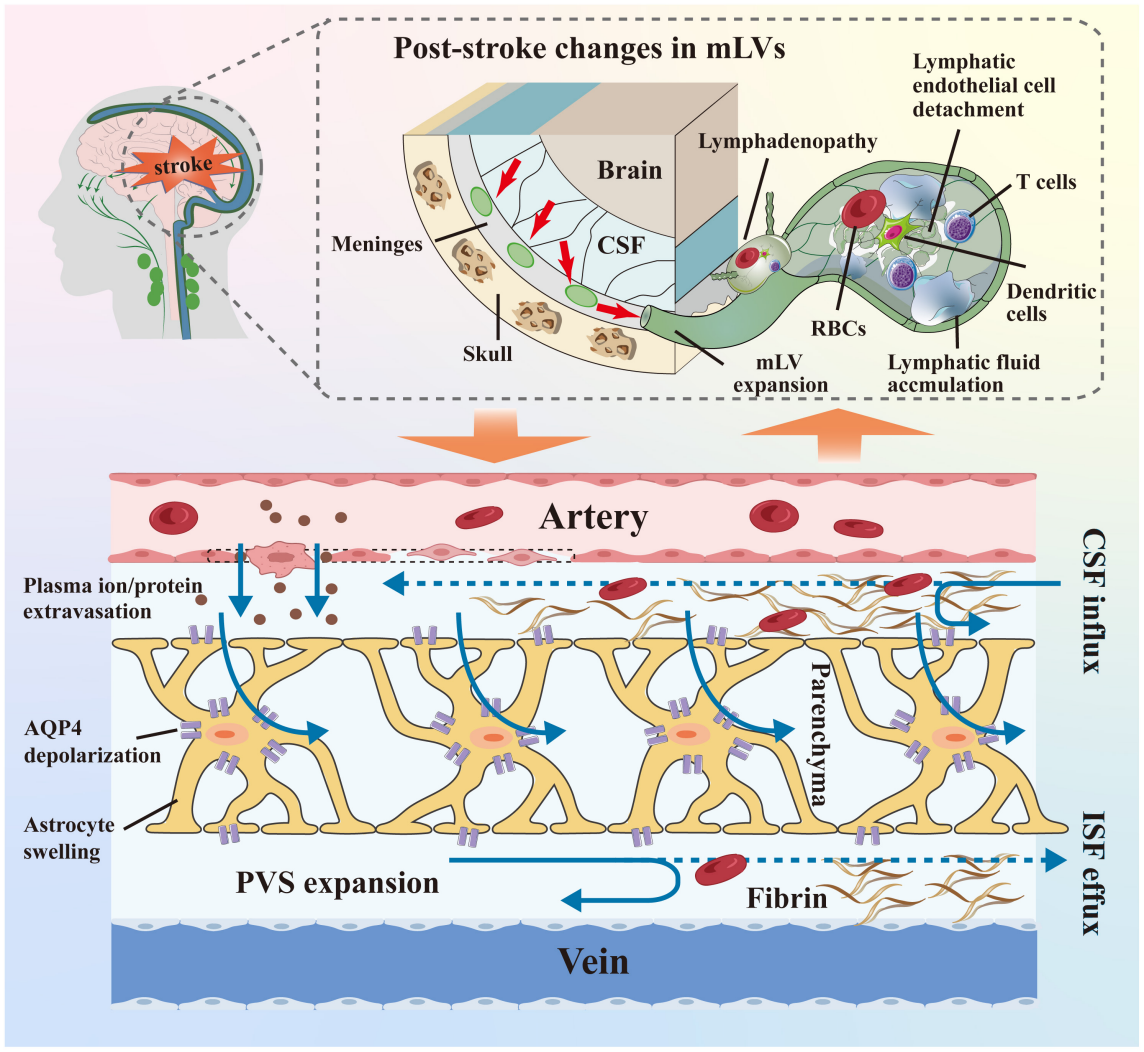
This diagram illustrates dysregulation of the glymphatic and meningeal lymphatic systems after stroke. Stroke injury may lead to BBB disruption, plasma ion/protein extravasation, AQP4 depolarization, astrocyte swelling, PVS expansion, and blood-product/fibrin deposition, thereby altering CSF influx and ISF efflux dynamics in a subtype- and stage-dependent manner. In the meningeal lymphatic system, mLVs may show pathological expansion or dysfunction, lymphatic fluid stagnation, and endothelial cell injury or detachment. T cells, dendritic cells, and red blood cells may accumulate in or around mLV-related drainage routes, further compromising lymphatic drainage. These alterations can contribute to impaired CSF drainage, lymphadenopathy, and secondary neuroinflammation, exacerbating post-stroke cerebral edema and injury. BBB, blood–brain barrier; AQP4, aquaporin-4; CSF, cerebrospinal fluid; PVS, perivascular spaces; ISF, interstitial fluid; mLVs, meningeal lymphatic vessels; RBCs, red blood cells.

Stroke impairs both glymphatic and meningeal lymphatic clearance, although the dominant abnormalities vary by stroke subtype and model. This section summarizes the major post-stroke changes in each pathway, with emphasis on subtype-specific mechanisms rather than imaging findings (Figure 2).

### 4.1 Glymphatic dysfunction after stroke

In ischemic stroke and experimental arterial occlusion models, one of the earliest abnormalities appears to be a rapid influx of CSF along perivascular routes into the injured hemisphere (Coupland et al., 2025; Mestre et al., 2020). Experimental work suggests that ischemic spreading depolarizations and associated vasoconstrictive changes can enlarge PVS and drive this transient fluid entry, thereby contributing to early tissue swelling (Mestre et al., 2020; Ji et al., 2021). As ischemia evolves, reduced arterial pulsatility, cytotoxic edema, and compression of periarterial and perivenous pathways are thought to impair physiological CSF-ISF exchange and slow glymphatic transport (Coupland et al., 2025; Han et al., 2023).

In ICH, glymphatic disturbance develops in a different microenvironment. A parenchymal hematoma and perihematomal edema create local mass effect, while hemoglobin, heme, iron, and other blood-derived products intensify oxidative and inflammatory injury in the surrounding tissue (Chen et al., 2021; Alsbrook et al., 2023). In this setting, perivascular routes may be compressed or secondarily distorted, and experimental work suggests that blocking cervical lymphatic drainage aggravates edema and neuroinflammation (Liu et al., 2021). Accordingly, clearance failure in ICH is more closely linked to perihematomal secondary injury than to the hyperacute CSF influx pattern described in ischemic stroke (Chen et al., 2021; Liu et al., 2021).

In SAH, blood enters the subarachnoid and CSF compartments directly (Lauzier et al., 2023). As a result, clot burden within CSF-containing spaces, elevated intracranial pressure, and prolonged exposure to erythrocyte degradation products can all interfere with glymphatic exchange (Lauzier et al., 2023; Goulay et al., 2017). Experimental studies indicate that glymphatic dysfunction may persist beyond the immediate hemorrhagic phase and may recover only partially over time, especially in large-animal models (Goulay et al., 2017; Wang et al., 2024; Pu et al., 2019).

Across stroke subtypes, disruption of the astrocyte endfoot-AQP4 interface provides an additional mechanistic link between vascular injury, edema, and impaired glymphatic exchange (Peng et al., 2023). Stroke-related BBB injury and neuroinflammation can damage the neurovascular interface, while altered AQP4 localization or polarization may reduce the efficiency of water and solute exchange along glymphatic routes (Huang et al., 2020; Candelario-Jalil et al., 2022; Alsbrook et al., 2023). Experimental ischemic stroke studies report altered AQP4 distribution, altered perivascular AQP4 expression, AQP4 depolarization, and disturbed CSF flow after ischemic injury (Mogoanta et al., 2014; Filchenko et al., 2020; Xin et al., 2023). Related work links glymphatic dysfunction to cerebral edema formation after ischemic stroke (Ji et al., 2021; Zhu et al., 2024). Potential downstream consequences include reduced ISF clearance, retention of proteinaceous and metabolic waste, amplification of neuroinflammation, and delayed secondary injury, although long-term clinical consequences remain less certain than experimental evidence (Cai et al., 2024; Goulay et al., 2020; Back et al., 2020).

### 4.2 Meningeal lymphatic dysfunction after stroke

In ischemic stroke, meningeal lymphatic alterations appear to be driven primarily by brain-to-CLNs immune signaling and dynamic VEGF-C/VEGFR3-dependent remodeling, rather than by direct clearance of blood (Esposito et al., 2019; Boisserand et al., 2024; Choi et al., 2025). After focal cerebral ischemia, the injured brain rapidly releases VEGF-C into the CSF and activates lymphatic endothelium and macrophage-related responses in CLNs, supporting an early brain-to-CLNs inflammatory axis (Esposito et al., 2019). Experimental studies further suggest that ischemia induces lymphatic remodeling near CSF egress routes, whereas developmental deficiency of meningeal lymphatics worsens stroke outcome (Yanev et al., 2020). However, the functional consequence of this response is phase-dependent and not uniformly beneficial. Pre-stroke enhancement of lymphatic drainage by VEGF-C can increase CSF outflow to dCLNs and attenuate peri-infarct inflammation (Boisserand et al., 2024). Similarly, VEGF-C-induced dural lymphatic growth has been associated with reduced astroglial reactivity and selected behavioral improvement despite no clear reduction in infarct or edema volume (Keuters et al., 2025). By contrast, acute post-stroke VEGF-C signaling may enlarge infarcts in some models (Choi et al., 2025), whereas blockade of meningeal lymphatic drainage can reduce early central and peripheral inflammatory responses (Yang L. et al., 2024). These apparently divergent findings suggest that, in ischemic stroke, meningeal lymphatics may either buffer tissue injury by facilitating fluid and antigen drainage or amplify acute neuroimmune signaling depending on timing, anatomical compartment, and experimental intervention. Beyond VEGF-C signaling, other experimental studies suggest that meningeal lymphatic modulation may influence post-ischemic repair and inflammation. Cranial bone transport improved neurological recovery in middle cerebral artery occlusion (MCAO) rats while enhancing angiogenesis, neurogenesis, and meningeal lymphatic drainage (Bai et al., 2022), whereas CCN1 blockade reduced reperfusion injury and spatial memory deficits by modulating detrimental inflammation involving CSF drainage, meningeal lymphatics, and CLNs (Lee et al., 2025).

In ICH, meningeal lymphatic function appears to be dominated by the clearance of hematoma-derived erythrocytes, iron, and other blood products, with experimental evidence showing that meningeal lymphangiogenesis and enhanced lymphatic drainage emerge during the late phase after ICH (Tsai et al., 2022). In contrast, ablation or blockade of mLVs impairs hematoma clearance, whereas pharmacological enhancement of lymphatic function reduces hematoma volume, residual erythrocytes, iron deposition, neuronal loss, and astroglial activation, while improving behavioral recovery (Tsai et al., 2022). Therefore, compared with ischemic stroke, the pathological relevance of meningeal lymphatics in ICH is more directly linked to blood-product removal and mitigation of secondary toxic injury.

In SAH, blood directly enters the subarachnoid space and burdens CSF drainage pathways, making meningeal lymphatic dysfunction earlier and more anatomically direct than in ischemic stroke. Meningeal lymphatics normally participate in the clearance of extravasated erythrocytes from the CSF to dCLNs after SAH (Chen et al., 2020), but experimental SAH causes marked and persistent impairment of glymphatic-meningeal drainage to dCLNs (Pu et al., 2019). Mechanistically, SAH can injure meningeal lymphatic endothelium through thrombospondin-1-CD47-related apoptotic signaling (Wang et al., 2023), while the meningeal microenvironment subsequently shifts from myeloid-cell-dominated acute inflammation to fibroblast-associated tissue remodeling; placental growth factor signaling may contribute to early repair of lymphatic structure and drainage (Zhu et al., 2025). Large-animal imaging studies further show that lymphatic outflow along the olfactory, optic, facial, and vestibulocochlear routes decreases profoundly within hours after SAH and improves over the following 1–2 weeks, whereas glymphatic function may recover only partially by 2 weeks (Wang et al., 2024). More recent experimental work further links SAH-induced meningeal lymphatic dysfunction to delayed cognitive impairment. In a mouse SAH model, mLV fragmentation and atrophy were associated with impaired CSF/ISF drainage, metabolite accumulation, hippocampal dysfunction, and progressive spatial learning and memory deficits, whereas VEGF-C-mediated activation of phosphoinositide 3-kinase-protein kinase B signaling preserved mLV function and alleviated cognitive impairment (Cai et al., 2026). Thus, SAH-related meningeal lymphatic dysfunction is best understood as a combination of subarachnoid blood burden, endothelial injury, impaired erythrocyte and immune-cell egress, and delayed structural remodeling.

Overall, the dominant pathological role of meningeal lymphatics differs across stroke subtypes: in ischemic stroke, they are closely linked to early brain-peripheral immune crosstalk and phase-dependent VEGF-C/VEGFR3 signaling; in ICH, they facilitate hematoma and iron clearance; and in SAH, they are directly challenged by subarachnoid blood, endothelial injury, and failure of CSF-borne erythrocyte drainage.

Taken together, available evidence indicates that stroke can disrupt both glymphatic and meningeal lymphatic pathways, but the timing, severity, and dominant mechanisms vary across ischemic stroke, ICH, and SAH and remain defined predominantly by experimental studies. In ischemic stroke, early perivascular CSF influx may be followed by impaired glymphatic exchange, whereas in ICH clearance failure is more directly linked to hematoma-derived toxicity and blood-product removal, and in SAH blood within CSF spaces directly burdens meningeal drainage pathways, with severe early outflow failure and only partial recovery in some preclinical models. These subtype-specific differences provide a rationale for multimodal imaging to monitor clearance-pathway dysfunction *in vivo*, although the clinical validity of current imaging biomarkers and the therapeutic value of clearance-targeted interventions remain to be established.

## 5 Applications of advanced MRI in assessing post-stroke glymphatic and lymphatic dysfunction

**Figure 3:**
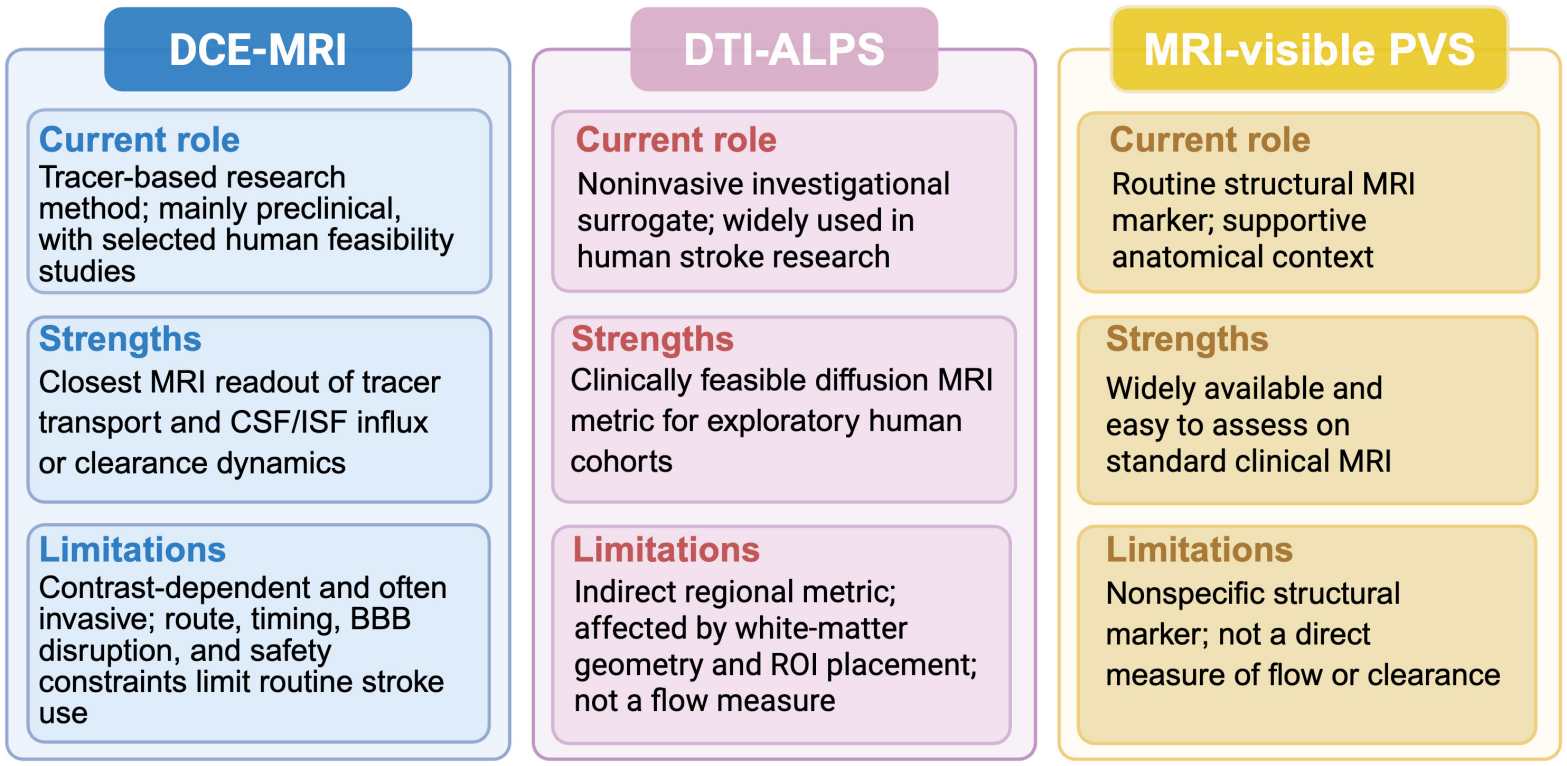
Comparison of three MRI readout classes used in post-stroke glymphatic and meningeal lymphatic research. DCE-MRI provides tracer-based information on CSF/ISF transport but is usually contrast-dependent, often invasive, and mainly supported by preclinical or selected feasibility studies. DTI-ALPS is a noninvasive diffusion MRI surrogate widely used in human stroke research, but it does not directly measure bulk flow or clearance. MRI-visible PVS provide routine structural context but are nonspecific and should not be interpreted as standalone evidence of glymphatic dysfunction. DCE-MRI, dynamic contrast-enhanced magnetic resonance imaging; DTI-ALPS, diffusion tensor image analysis along the perivascular space; PVS, perivascular spaces; CSF, cerebrospinal fluid; ISF, interstitial fluid. Created in BioRender. Jin, Y. (2026) https://BioRender.com/k4kqr3j.

MRI has enabled *in vivo* investigation of post-stroke clearance dysfunction, but available readouts differ markedly in what they sample, how directly they reflect glymphatic or meningeal lymphatic transport, and how mature the supporting evidence is. The techniques below are therefore organized by biological target and evidentiary maturity, with direct tracer-based methods distinguished from indirect MRI surrogates and human data separated from experimental evidence (Figure 3). The imaging methods reviewed below differ substantially in biological directness. Tracer-based DCE-MRI provides relatively direct evidence of CSF/ISF transport but is often invasive or contrast-dependent. DTI-ALPS and MRI-visible PVS are more feasible in human studies but remain indirect surrogate markers. Therefore, associations between these imaging readouts and molecular mechanisms such as AQP4 polarization, VEGF-C/VEGFR3 signaling, or inflammatory lymphatic remodeling should be interpreted as mechanistic hypotheses unless directly supported by paired molecular and imaging data.

### 5.1 MRI methodologies for imaging glymphatic and lymphatic function

#### 5.1.1 Dynamic contrast-enhanced MRI

DCE-MRI remains one of the most direct MRI approaches for studying post-stroke clearance because it tracks tracer movement through CSF spaces and into brain tissue (Zhu et al., 2023; Klostranec et al., 2021; Gaberel et al., 2014). In experimental ischemic stroke, tracer-based MRI has shown impaired glymphatic perfusion after stroke and suppressed glymphatic transport kinetics after ischemic edema formation, consistent with disturbed perivascular transport (Gaberel et al., 2014; Zhang J. et al., 2022). Human DCE-MRI work has demonstrated that parasagittal dural enhancement related to meningeal lymphatic pathways can be visualized (Joo et al., 2023). These human studies support anatomical feasibility rather than stroke-specific clinical validation. However, most stroke applications remain preclinical and commonly rely on intrathecal or intracisternal contrast administration. Interpretation is also influenced by tracer route, timing, BBB status, and the safety constraints of exogenous contrast agents (Zhu et al., 2023; Zhang J. et al., 2022; Bhave et al., 2008; Gulani et al., 2017).

#### 5.1.2 Diffusion tensor image analysis along the perivascular space

DTI-ALPS is the most widely used noninvasive MRI surrogate in human stroke studies (Qin et al., 2023; Zhang C. et al., 2022). It estimates water diffusivity along presumed perivascular directions in deep white matter and has been applied in both subacute ischemic stroke and spontaneous ICH (Qin et al., 2023; Zhang C. et al., 2022; Taoka et al., 2024). Lower ALPS indices have been reported on the affected side, with exploratory associations with motor, cognitive, prognostic, or disease-duration measures in selected studies (Xia et al., 2024; Qin et al., 2023; Zhang C. et al., 2022; Zeng et al., 2025; Wang Y. et al., 2025). However, DTI-ALPS does not measure bulk flow or whole-brain clearance and is sensitive to white-matter geometry, region-of-interest placement, and acquisition parameters (Taoka et al., 2024; Ringstad, 2024; Schilling et al., 2025). It should therefore be described as an indirect regional surrogate rather than an established clinical biomarker of post-stroke glymphatic dysfunction.

#### 5.1.3 Perivascular space imaging

PVS imaging is not a flow measure but a structural marker that is widely available on routine MRI (Wardlaw et al., 2020). Enlarged PVS burden may accompany vascular injury, abnormal fluid handling, and neuroinflammatory or BBB-related processes, but it is biologically nonspecific and should not be equated directly with glymphatic failure (Wardlaw et al., 2020; Debette et al., 2019; Hong et al., 2025). In stroke-related settings, PVS changes have been described in a subtype- and region-dependent manner, including aneurysmal SAH (Yu et al., 2024). PVS imaging can therefore complement diffusion- or tracer-based methods, but it is better interpreted as supportive structural evidence than as a standalone marker of clearance function.

#### 5.1.4 Other MRI approaches

Phase-contrast MRI and time-spatial labeling inversion pulse provide information on CSF pulsatility or tagged CSF motion, but neither directly measures glymphatic clearance (Spijkerman et al., 2019; Yamada et al., 2015). Intravoxel incoherent motion and magnetization transfer indirect spin labeling offer additional information on slow fluid motion or CSF-tissue water exchange, yet their relevance to post-stroke clearance remains indirect and largely exploratory (Yamada et al., 2024, 2023; Li et al., 2022; Chen Z. et al., 2024). Chemical exchange saturation transfer provides metabolic or pH-related context rather than a direct measure of glymphatic transport (Wang et al., 2025; Consolino et al., 2020). These methods should be presented as adjunctive techniques rather than as validated stroke biomarkers.

### 5.2 Major MRI findings on glymphatic dysfunction after stroke

Because stroke encompasses heterogeneous pathological entities, we summarize MRI findings on post-stroke glymphatic dysfunction separately for ischemic stroke, ICH, and SAH. Across these subtypes, the directness of MRI evidence varies substantially, with the most direct findings coming from tracer-based studies, whereas most human studies still rely on indirect surrogate markers such as DTI-ALPS (Table 1, Supplementary Table S1).

**Table 1:** Studies investigating the glymphatic system in stroke.

References	Evidence type/directness	Population and sample size	Stroke subtype and timing	Protocol/readout	Outcome and reported effect/direction	Key limitation/interpretation
Human studies—ischemic stroke/cerebral infarction						
Toh and Siow (2021)	Human indirect MRI surrogate	50 ischemic stroke patients; 44 healthy controls.	Ischemic stroke; MRI 1–60 days after onset (mean 17.1 days).	DTI-ALPS; bilateral and infarct/contralateral comparison.	Ipsilateral ALPS was lower than contralateral ALPS and increased with time since onset.	Cross-sectional; variable timing and lesion size; DTI-ALPS is indirect.
Qin et al. (2023)	Human indirect MRI surrogate	20 analyzed subacute IS patients with single left subcortical lesions; 30 controls.	Subacute ischemic stroke; DTI 7–40 days after onset.	DTI-ALPS; Fugl-Meyer motor/sensory scores; CST DTI metrics.	Lower left ALPS in stroke; higher ALPS correlated with better motor score.	Small left-lesion-only cohort; no longitudinal or tracer validation.
Zeng et al. (2025)	Human prospective indirect MRI surrogate	59 AIS patients; 29 matched controls; 18 had follow-up MRI.	AIS; baseline MRI 5.9 ± 3.6 days; follow-up about 62 days.	DTI-ALPS; FMA-UE, NIHSS, infarct volume, follow-up change.	Ipsilateral ALPS was lower and associated with FMA-UE/NIHSS; ALPS increased at follow-up.	Single-center; limited follow-up scans; DTI-ALPS does not measure whole-brain flow.
Wang Y. et al. (2025)	Human prospective indirect MRI surrogate	29 acute subcortical infarct patients; 25 controls after exclusions.	Acute subcortical infarction within 72 h; MoCA at 7 and 90 days.	3.0-T DTI-ALPS; MoCA; ROC analysis for early PSCI.	Bilateral ALPS was lower than controls; lesion-side ALPS correlated with early MoCA.	Small single-center cohort; restricted infarct subtype; specificity limited.
Zhu et al. (2026)	Human acute-phase indirect MRI surrogate	69 AIS patients; 34 healthy controls.	First-ever unilateral basal ganglia/corona radiata AIS; MRI within 72 h.	Automated DTI-ALPS; NIHSS and FMA-UE with laterality analysis.	Left ALPS was reduced in AIS and related to early motor/neurological status.	Cross-sectional acute-only study; lateralization mechanism uncertain.
Finkelstein et al. (2025)	Human post-treatment indirect MRI surrogate	55 analyzable anterior-circulation LVO patients after thrombectomy.	AIS after thrombectomy; early post-treatment MRI; 90-day outcome.	Post-thrombectomy DTI-ALPS-derived glymphatic metric.	Infarct-side metric was lower than contralateral side; lower values associated with futile recanalization.	Highly selected thrombectomy cohort; indirect metric; observational association.
Umehana et al. (2026)	Human exploratory meningeal-lymphatic MRI evidence	11 recent cerebral infarction/cerebrovascular-disorder patients; 6 moyamoya disease patients.	Recent cerebral infarction; delayed post-contrast MRI 1–6 h after GBCA.	Delayed DANTE T1-SPACE vessel-wall MRI; meningeal/dural enhancement.	All five large infarcts had ipsilateral delayed meningeal enhancement; none of 6 small infarcts did.	Very small exploratory cohort; delayed enhancement is an indirect clearance marker.
Zhu et al. (2024)	Mixed translational evidence	Mouse MCAO experiments plus 18 adult AIS patients after endovascular treatment; 10 had repeat DTI.	Animal transient MCAO in first week; human DTI at 24 ± 4 h and subset at 72 ± 12 h.	Animal tracer/imaging and glymphatic modulation; human DTI-ALPS with edema/outcome.	Glymphatic dysfunction tracked edema; lower human ALPS related to edema severity/outcome.	Small human component; mixed preclinical/clinical evidence; DTI-ALPS is indirect.
Human studies—intracerebral hemorrhage						
Zhang C. et al. (2022)	Human indirect MRI surrogate	20 left-hemisphere spontaneous ICH patients; 31 controls.	Subacute/chronic mixed ICH; disease duration 21–155 days.	DTI/SWI; lesion-side and nonlesion-side DTI-ALPS.	Lesion-side ALPS was reduced and correlated positively with disease duration.	Small sample; left-hemisphere ICH only; manual ROI; no outcome analysis.
Xia et al. (2024)	Human exploratory indirect MRI surrogate	58 mild-to-moderate spontaneous ICH patients; 63 matched controls.	Acute-subacute and chronic subgroup analyses; 90-day outcome.	DTI-ALPS plus hematoma/edema and MoCA assessment.	Lower ALPS in ICH; ALPS related to hematoma volume, cognition, and poor 90-day outcome.	Single-center retrospective study; mild-to-moderate ICH only; needs validation.
Cai et al. (2025)	Human acute ICH indirect MRI surrogate	55 acute spontaneous ICH patients; 97 matched controls.	Acute spontaneous ICH; 90-day mRS outcome.	DTI-ALPS with hemorrhage and edema volume assessment.	Ipsilateral ALPS was lower than controls and associated with hemorrhage/edema burden; higher ALPS predicted favorable outcome.	Observational acute cohort; hemorrhage/edema may confound diffusion metrics.
Human studies—subarachnoid hemorrhage						
Eide et al. (2025)	Human direct tracer-based MRI	27 SAH patients; 22 reference subjects.	Post-SAH time-evolution study: < 3, 3–6, 6–12, and >12 months.	Intrathecal gadobutrol-enhanced MRI; tracer enrichment and redistribution.	SAH impaired glymphatic enrichment and redistributed CSF tracer toward ventricles.	Invasive intrathecal protocol; small subgroups; heterogeneous timing.
Oka et al. (2026)	Human indirect MRI surrogate	60 aneurysmal SAH patients.	DTI at median 18 days; 3-month outcome and shunt-dependent hydrocephalus.	DTI-ALPS; mediation analysis.	Poor-outcome patients had lower ALPS; low ALPS was associated with poor outcome partly via hydrocephalus.	Single-center retrospective study; DTI-ALPS is indirect; mediation is not causality.
Lu et al. (2026)	Human multimodal MRI surrogate	58 acute SAH, 26 chronic post-SAH hydrocephalus, 41 HCs; glymphatic MRI *n* = 57/41; mLV-dCLN MRI *n* = 58/41; chronic hydrocephalus *n* = 22–24 by sequence.	Spontaneous SAH; acute and chronic hydrocephalus-related analyses.	DTI-ALPS, FW-WM, PVSVF-WM; mLV and dCLN signal intensity.	SAH showed reduced ALPS and reduced mLV/dCLN signal; mLV signal loss predicted shunt-dependent hydrocephalus.	Indirect MRI surrogates; single-center exploratory cohort; mLV and dCLN signal intensities are not direct lymphatic-flow measurements.
Experimental studies—ischemic or mixed stroke models						
Mestre et al. (2020)	Experimental direct physiological imaging/tracer evidence	Male rodent focal-ischemia/MCAO experiments; 3D-FIESTA MRI *n* = 7 sham/8 MCAO; AQP4 assays *n* = 4–6/group.	Hyperacute ischemic stroke; minutes-to-hours after arterial occlusion/spreading depolarization.	MRI, radiolabeled tracers, and multiphoton imaging of perivascular CSF influx.	CSF rapidly entered ischemic tissue and contributed to early swelling; direction was increased influx/edema.	Preclinical hyperacute physiology; not a validated clinical clearance biomarker.
Gaberel et al. (2014)	Experimental direct tracer-based MRI	Mouse experimental stroke models; key MRI quantification *n* = 5/group.	Experimental stroke models; early post-stroke 3-h/24-h windows.	Contrast-enhanced MRI after CSF-space gadolinium administration.	Stroke reduced glymphatic perfusion; fibrinolysis partially restored perfusion in the experimental setting.	Small preclinical groups; mixed stroke contexts; not standardized clinical MRI.
Zhang J. et al. (2022)	Experimental direct tracer-based MRI	Male C57BL/6 mice; MRI *n* = 3/group; pathology/AQP4 *n* = 4/group.	Permanent MCAO with intact BBB; hyperacute MRI after occlusion.	Intracisternal BOPTA-Gd; 11.7-T T1WI tracer kinetics with DWI/ADC and AQP4 assessment.	Ipsilateral cortical/striatal tracer uptake was reduced, contralateral uptake increased, and bilateral ventricular influx was reduced/delayed; acute ischemic edema was associated with suppressed glymphatic transport.	Very small MRI sample; high-field preclinical protocol; permanent occlusion model.
Warren et al. (2023)	Experimental fluorescent CSF-tracer transport assay	Photothrombotic stroke mice; assay-specific n by tissue/time point.	Photothrombotic ischemic stroke; 24 h and 2 weeks.	Fluorescent CSF tracer influx into brain and outflow to nasal mucosa.	CSF tracer influx and nasal outflow were reduced at 24 h but not at 2 weeks.	Animal-only tracer endpoint; attrition; not an MRI biomarker.
Lin et al. (2020)	Experimental direct tracer-based MRI	12 Sprague-Dawley rats: sham *n* = 4, MCAO 1 day *n* = 4, MCAO 7 days *n* = 4.	Left MCAO; imaging at 1 or 7 days.	Intrathecal Gd-DTPA; 3D T1-weighted MRI over 6 h.	Remote degeneration regions showed abnormal tracer kinetics in ipsilateral substantia nigra and ventral thalamus.	Very small groups; evaluates remote degeneration rather than infarct core.
Li C. et al. (2022)	Experimental longitudinal direct tracer-based MRI plus pathology	20 adult male Sprague-Dawley rats: sham and MCAO 1 week/2 weeks/2 months, *n* = 5 each.	Transient MCAO; DCE-MRI and pathology at 1 week, 2 weeks, and 2 months.	3.0-T intrathecal Gd-DTPA DCE-MRI; GFAP, AQP4, Iba1, APP.	Thalamic glymphatic impairment and APP-related pathology were most evident at 2 weeks.	Small groups; thalamic secondary-region readout; incomplete MRI-histology correlation.
Experimental studies—intracerebral hemorrhage						
Liu et al. (2021)	Experimental mechanistic intervention evidence	75 rats: sham *n* = 25, ICH *n* = 25, ICH + CLB *n* = 25.	Collagenase-induced ICH; mainly 3 and 7 days after ICH.	Cervical lymphatic blockade; edema, cytokines, AQP4, apoptosis, neurological score.	Drainage blockade aggravated edema, neuroinflammation, apoptosis, and neurological deficits.	Animal-only intervention; blockade may have off-target effects; not a human biomarker study.
Experimental studies—subarachnoid hemorrhage						
Goulay et al. (2017)	Experimental large-animal direct CSF-circulation evidence	Three adult cynomolgus monkeys.	Baseline and acute post-SAH assessment.	MRI tracking of CSF tracer/contrast distribution.	SAH severely impaired brain parenchymal CSF circulation.	Very small nonhuman primate sample; invasive protocol; no clinical threshold.
Wang et al. (2024)	Experimental large-animal direct tracer-based MRI	14 male beagles: sham *n* = 3, SAH-1d *n* = 3, SAH-2w *n* = 4, SAH + CSF drain *n* = 4.	Beagle SAH; MRI before SAH and at 1 h, 1 week, and 2 weeks.	Cisterna magna Gd-DTPA with serial T1 MRI; glymphatic and mLV outflow analysis.	SAH caused early glymphatic and meningeal lymphatic outflow impairment with partial recovery by 2 weeks; CSF drainage improved readouts.	Preclinical; small groups; invasive intrathecal contrast protocol.
Pu et al. (2019)	Experimental biological support; non-MRI	Three-month-old male C57BL/6 mice in sham and SAH groups; tracer/histology assays commonly *n* = 4/group; behavioral assays *n* = 8/group.	Cisterna-magna blood-injection SAH; mainly 1 day and 1 week readouts.	Fluorescent tracer drainage, histology, AQP4, inflammation, and behavior.	SAH impaired glymphatic influx and meningeal lymphatic drainage to deep cervical lymph nodes.	Non-MRI animal study; small assay-specific sample sizes; mechanistic support only.

This table summarizes published studies across human and animal stroke models, highlighting imaging techniques, molecular assessments, outcome measures, and major limitations.

3D, three-dimensional; 3D-FIESTA, three-dimensional fast imaging employing steady-state acquisition; ADC, apparent diffusion coefficient; AIS, acute ischemic stroke; ALPS, analysis along the perivascular space; APP, amyloid precursor protein; AQP4, aquaporin-4; BBB, blood–brain barrier; BOPTA-Gd, gadobenate dimeglumine; CLB, cerebral lymphatic blockade; CSF, cerebrospinal fluid; CST, corticospinal tract; DANTE, delay alternating with nutation for tailored excitation; dCLN, deep cervical lymph node; DCE-MRI, dynamic contrast-enhanced magnetic resonance imaging; DTI, diffusion tensor imaging; DTI-ALPS, diffusion tensor image analysis along the perivascular space; DWI, diffusion-weighted imaging; FMA-UE, Fugl–Meyer Assessment of the Upper Extremity; FW-WM, free water in white matter; GBCA, gadolinium-based contrast agent; Gd-DTPA, gadolinium–diethylenetriaminepentaacetic acid (gadopentetate dimeglumine); GFAP, glial fibrillary acidic protein; HCs, healthy controls; Iba1, ionized calcium-binding adaptor molecule 1; ICH, intracerebral hemorrhage; IS, ischemic stroke; LEC, lymphatic endothelial cell; LVO, large-vessel occlusion; MCAO, middle cerebral artery occlusion; mLV, meningeal lymphatic vessel; MoCA, Montreal Cognitive Assessment; MRI, magnetic resonance imaging; mRS, modified Rankin Scale; NIHSS, National Institutes of Health Stroke Scale; PI3K-AKT, phosphoinositide 3-kinase–protein kinase B signaling pathway; PSCI, post-stroke cognitive impairment; PVSVF-WM, white matter perivascular space volume fraction; RNA-seq, ribonucleic acid sequencing; ROC, receiver operating characteristic; ROI, region of interest; SAH, subarachnoid hemorrhage; SWI, susceptibility-weighted imaging; T1-SPACE, T1-weighted sampling perfection with application-optimized contrasts using different flip-angle evolutions; T1WI, T1-weighted imaging; VEGF-C, vascular endothelial growth factor C; VEGFR3, vascular endothelial growth factor receptor 3.

In ischemic stroke, direct MRI evidence comes mainly from experimental tracer-based studies. DCE-MRI in permanent middle cerebral artery occlusion (pMCAO) mice has shown early impairment of glymphatic transport after ischemic edema (Zhang J. et al., 2022), and another mouse study found that CSF tracer entry into brain parenchyma and outflow to the nasal mucosa were reduced at 24 h but not at 2 weeks after stroke (Warren et al., 2023). DCE-MRI studies in rats further suggest glymphatic impairment in secondary degeneration areas, including the ipsilateral substantia nigra and ventral thalamic nucleus at 1 day and 7 days after MCAO, and prominent thalamic impairment around 2 weeks after MCAO (Lin et al., 2020; Li C. et al., 2022). In humans, the evidence is less direct and is based mainly on DTI-ALPS. Human DTI-ALPS studies in ischemic stroke have reported lower ALPS values on the infarct side than on the contralateral side or in controls, with associations with motor dysfunction in subacute and acute-stage cohorts (Qin et al., 2023; Toh and Siow, 2021; Zhu et al., 2026). In large-vessel occlusion ischemic stroke treated with mechanical thrombectomy, a post-thrombectomy DTI-ALPS-derived glymphatic metric was lower in patients with futile recanalization than in those with 90-day functional independence, and the ALPS index correlated inversely with baseline National Institutes of Health Stroke Scale (NIHSS) score (Finkelstein et al., 2025). Taken together, MRI findings in ischemic stroke support early and spatially heterogeneous glymphatic dysfunction, but most human evidence remains indirect and should not be interpreted as a direct measure of whole-brain clearance.

In ICH, the MRI literature is narrower and is dominated by human DTI-ALPS studies rather than direct tracer-based glymphatic imaging (Cai et al., 2025). Available studies report lower ipsilateral ALPS values in spontaneous ICH, with disease-duration associations in one study (Zhang C. et al., 2022). More recent exploratory data suggest associations with hematoma volume, a trend toward association with perihematomal edema volume that did not remain significant after false discovery rate correction, and 90-day functional outcome (Xia et al., 2024). These observations are compatible with impaired perihematomal clearance, but because DTI-ALPS is an indirect white-matter diffusion metric, current MRI evidence in ICH should be framed as suggestive of glymphatic dysfunction rather than as a direct demonstration of impaired fluid transport.

SAH should be discussed separately because blood enters the subarachnoid CSF compartment directly. Here the MRI evidence is both more anatomically relevant and somewhat more direct. In beagle SAH, serial T1-weighted MRI after intrathecal gadolinium-diethylenetriamine pentaacetic acid (Gd-DTPA) showed reduced CSF influx and delayed or reduced lymphatic outflow within hours to 1 week, with better restoration of lymphatic function and partial glymphatic recovery by 2 weeks (Wang et al., 2024). Human data have also recently emerged. A prospective intrathecal contrast-enhanced MRI study found marked impairment of glymphatic enrichment after SAH, together with redistribution of CSF tracer from the subarachnoid space toward the ventricles, and the impairment appeared greatest in the earlier post-hemorrhage period (Eide et al., 2025). In that study, alterations appeared most pronounced in the 3–6 month subgroup and less pronounced after 12 months, although time-course interpretation should remain cautious because the subgroup sizes were small (Eide et al., 2025). In aneurysmal SAH patients, the DTI-ALPS index was significantly reduced in those with poor 3-month outcomes and shunt-dependent hydrocephalus, suggesting an association between impaired glymphatic clearance and adverse prognosis after SAH (Oka et al., 2026). Accordingly, SAH is currently the stroke subtype in which MRI most directly visualizes clearance-pathway failure *in vivo*, although the human literature remains small and invasive-tracer protocols are not yet routine.

Across all stroke subtypes, MRI markers differ substantially in biological directness. Intrathecal contrast-enhanced MRI is the most direct MRI approach for assessing human perivascular tracer transport, but its invasiveness limits routine use. DTI-ALPS is easier to implement clinically, yet it reflects local diffusion behavior in deep white matter rather than bulk CSF-ISF flow or clearance rate. Moreover, DTI-ALPS can be confounded by white-matter geometry and crossing fibers, and a recent comparison with intrathecal contrast-enhanced MRI found only limited correspondence between ALPS and tracer-based glymphatic measures. MRI-visible PVS burden may provide supportive regional information, but it is not specific to glymphatic dysfunction because enlarged PVS likely have multiple overlapping etiologies.

### 5.3 MRI insights into meningeal lymphatic dysfunction after stroke

Compared with glymphatic imaging, MRI evidence for post-stroke meningeal lymphatic dysfunction remains limited. Human non-stroke MRI studies have nevertheless established that meningeal lymphatic pathways can be visualized *in vivo* (Joo et al., 2023). Non-contrast 3D T2-FLAIR has reported visualization of dorsal and ventral dural lymphatic pathways with connections to dCLNs (Albayram et al., 2022). Intravenous DCE-MRI of the parasagittal dural space has shown a slow enhancement and delayed wash-out pattern distinct from adjacent venous structures (Joo et al., 2023). These studies provide the imaging framework for stroke applications, but they do not by themselves validate stroke-specific biomarkers (Table 2, Supplementary Table S2).

**Table 2:** Studies investigating the meningeal lymphatic system in stroke.

References	Evidence type/directness	Population and sample size	Stroke subtype and timing	Protocol/readout	Outcome and reported effect/direction	Key limitation/interpretation
Human studies—ischemic stroke/cerebral infarction						
Umehana et al. (2026)	Human exploratory indirect mLV imaging	11 patients with recent cerebral infarction and 6 patients with moyamoya disease.	Subacute cerebral infarction; MRI performed 11.5 ± 4.5 days after onset; delayed imaging 1–6 h after GBCA administration.	Whole-brain DANTE T1-SPACE/VW-MRI before, immediately after, and 1–6 h after contrast	All 5 large infarctions showed ipsilateral delayed meningeal enhancement; 0/6 small infarctions did.	Very small sample; delayed enhancement is indirect and confounded by infarct size, BBB disruption, and contrast timing.
Human studies—subarachnoid hemorrhage						
Lu et al. (2026)	Human exploratory multimodal MRI surrogate	58 acute SAH, 26 chronic post-SAH hydrocephalus, 41 healthy controls; glymphatic MRI *n* = 57/41; mLV-dCLN MRI *n* = 58/41; chronic hydrocephalus *n* = 22–24 by sequence.	Clinical SAH; chronic shunt-dependent hydrocephalus subgroup	DTI-ALPS, FW-WM/PVSVF-WM, mLV signal, and dCLN signal	SAH showed reduced ALPS and lower mLV/dCLN signals; abnormalities were worse in chronic hydrocephalus; mLV signal loss predicted shunt dependence.	Indirect MRI surrogates; single-center exploratory cohort; mLV and dCLN signal intensities are not direct lymphatic-flow measurements.
Experimental studies—ischemic stroke						
Esposito et al. (2019)	Experimental brain-CLN signaling evidence	Male Sprague-Dawley rats and male C57BL/6 mice. Key n: rat CLN IHC *n* = 4/group; rat FACS sham *n* = 3, MCAO *n* = 11, MCAO + MAZ51 *n* = 7; mouse MAZ51 infarct MCAO *n* = 8/MCAO + MAZ51 *n* = 9; CLN-removal infarct control *n* = 10/removal *n* = 11; ILN-removal control *n* = 7/removal *n* = 6.	Early post-ischemic phase	CSF/CLN VEGF-C/VEGFR3 assays, immune profiling, CLN/VEGFR3 interventions	Ischemia activated VEGF-C/VEGFR3 brain-to-CLN lymphatic-immune signaling; pathway blockade reduced inflammatory injury.	Mechanistic preclinical evidence; not a clinical imaging biomarker.
Yanev et al. (2020)	Experimental causal/developmental model	Adult male C57BL/6, Prox1-tdTomato, and Vegfr3 wild-type/mutant mice; PT surgeries *n* = 27; tMCAO surgeries *n* = 34 attempted; figure-level n typically PT *n* = 5–6 vs. sham *n* = 4 and tMCAO *n* = 5–7 vs. sham *n* = 5.	Experimental ischemic stroke; acute/subacute outcome	Stroke in mice with impaired mLV development	PT, but not tMCAO, induced mLV growth; impaired mLV development increased infarct volume after tMCAO, but not PT.	Developmental deficiency may not reflect acute adult therapeutic modulation.
Yang J. et al. (2024)	Experimental intervention evidence	Male Sprague–Dawley rats; 90 rats randomized to dCLN removal + MCAO, AAV-VEGF-C + MCAO, and MCAO groups.	Acute post-ischemic phase	Cerebral lymphatic blockade with central/peripheral inflammatory readouts	Blocking cerebral lymphatic drainage reduced central and peripheral inflammatory responses.	Shows timing-dependent and bidirectional lymphatic effects; not an imaging-biomarker study.
Boisserand et al. (2024)	Experimental prophylactic intervention evidence	Male C57BL/6J and Vegfr3::YFP mice; Key assays: CSF-MRI/DCE-MRI *n* = 10/group (20 mice); dural LEC sorting *n* = 30 mice/group; brain snRNA-seq *n* = 5/group.	Pre-stroke VEGF-C prophylaxis and early/subacute post-stroke readouts	AAV-VEGF-C delivery; drainage to dCLNs, inflammation, motor outcome	VEGF-C prophylaxis increased CSF lymphatic drainage and modulated neuroinflammation with improved recovery.	Prophylactic design limits clinical translation; timing likely critical.
Keuters et al. (2025)	Experimental interventional MRI evidence	Young adult male C57BL/6 mice; tMCAO;VEGF-C *n* = 50, sham;VEGF-C *n* = 35, tMCAO;control *n* = 49, sham;control *n* = 34; Gd-MRI cohort *n* = 7/6/6/6 after exclusions.	tMCAO; AAV9-VEGF-C administered 14 or 35 days before stroke; outcomes assessed at 1, 3, and 7 days after stroke.	Gd-enhanced MRI of CSF/ISF drainage and cribriform outflow; behavior/histology	VEGF-C-induced dural lymphatic growth increased CSF/ISF drainage and improved selected behavioral/histologic readouts.	Treatment-linked preclinical readout; does not define a spontaneous clinical mLV imaging phenotype.
Choi et al. (2025)	Experimental timing-dependent intervention evidence	10–12-week-old male mice; *n* = 8 sham and *n* = 9 tMCAO per time point; dCLN *n* = 6/group; CSF MRI *n* = 3 sham/5 tMCAO; MAZ51 *n* = 6/group; VEGF-C156S *n* = 3–4/group.	Acute and later post-stroke phases	VEGF-C/VEGFR3 manipulation; lymphangiogenesis, infarct, immune outcomes	VEGFR3 inhibition improved early outcomes, whereas acute VEGF-C delivery enlarged infarcts; effects were phase-dependent.	Important caution: mLV/VEGF-C modulation is not uniformly beneficial; timing is critical.
Bai et al. (2022)	Experimental recovery intervention evidence	136 male Sprague-Dawley/GFP rats total; *n* = 4–7/group for behavior, infarct, and perfusion; *n* = 4–5/group for angiogenesis/neurogenesis; *n* = 5/group for mLV drainage; *n* = 6/group for T-cell infiltration.	Subacute recovery phase	Cranial bone transport with lymphatic, angiogenic, neurogenic, and behavioral readouts	Cranial bone transport enhanced mLV drainage and recovery-related markers; lesions and neurological deficits were reduced.	Complex surgical intervention; translational feasibility uncertain; mainly mechanistic.
Lee et al. (2025)	Experimental brain-CLN inflammatory mechanism	235 male C57BL/6JNarl mice total; Key assays: TTC infarct *n* = 3–6/group; scRNA-seq *n* = 4/group; CCN1 treatment/blockade assays *n* = 3–7/group.	Early reperfusion and later behavioral outcomes	mLV disruption, scRNA-seq/inflammatory-cell analyses, CCN1 blockade	CCN1-mediated brain-CLN inflammation worsened injury; CCN1 blockade reduced inflammation and deficits.	Mechanistic rather than imaging-biomarker evidence.
Experimental studies—intracerebral hemorrhage						
Tsai et al. (2022)	Experimental ICH causal drainage evidence	294 male C57BL/6 mice total; assay-specific *n* = 3–23/group: mLV morphology *n* = 6–8/time point, drainage *n* = 3–6/time point, hematoma *n* = 5–9/group, cilostazol behavior *n* = 22–23.	Assessments were performed from the acute phase through 60 days after ICH.	mLV manipulation; drainage assays; hematoma, iron, histology, behavior	Enhancing mLV function accelerated hematoma/iron clearance and recovery; ablation/blockade worsened clearance.	Animal-only; dedicated human MRI biomarkers for ICH-related mLV dysfunction remain sparse.
Experimental studies—subarachnoid hemorrhage						
Chen et al. (2020)	Experimental direct erythrocyte-clearance evidence	Mouse SAH model. Key assays: dCLN erythrocyte drainage control/saline *n* = 12 and SAH *n* = 11; meningeal erythrocyte drainage control/saline *n* = 13 and SAH *n* = 15; CFSE-RBC tracing: meningeal analysis *n* = 12 mice/group; nodal analyses *n* = 12–23 lymph nodes/group; ablation/VEGFR3/behavior assays *n* = 5–22/group.	Post-SAH erythrocyte clearance phase	Tracking extravasated erythrocytes from CSF/subarachnoid space to mLVs and dCLNs	mLVs cleared SAH-derived erythrocytes into dCLNs; impaired drainage aggravated SAH injury.	Mouse model; focuses on blood-product drainage rather than clinical MRI.
Pu et al. (2019)	Experimental biological support; non-MRI	Three-month-old male C57BL/6 mice; tracer/histology assays commonly *n* = 4/group; behavioral assays *n* = 8/group.	Mainly 1 day and 1 week after SAH	Fluorescent tracer distribution, histology, AQP4, inflammation, behavior	SAH caused persistent glymphatic and mLV drainage malfunction.	Non-MRI animal study; supports mechanism but not biomarker readiness.
Wang et al. (2023)	Experimental molecular/spatial mechanism	Male C57BL/6 mice. Main scRNA-seq: 30 mice total; key validation: dCLN bead drainage *n* = 8/group; behavioral tests *n* = 10–12/group; human CSF/prognosis association *n* = 48.	Post-SAH meningeal injury and repair phases	scRNA-seq, spatial transcriptomics, endothelial injury/apoptosis assays	SAH injured mLV endothelium; THBS1-CD47-related apoptotic signaling was implicated.	Mechanistic omics evidence; not an imaging readout.
Zhu et al. (2025)	Experimental molecular/time-course mechanism	117 young male C57BL/6 mice total	Acute inflammation to subacute remodeling after SAH	Spatiotemporal profiling of meningeal microenvironment and mLV function	Meningeal environment shifted from inflammation to remodeling; PGF signaling may support early mLV repair.	Preclinical mechanistic study; clinical imaging counterpart not established.
Wang et al. (2024)	Experimental large-animal direct tracer-based MRI	14 male beagles: sham *n* = 3, SAH day 1 *n* = 3, SAH week 2 *n* = 4, SAH + CSF drain *n* = 4	Beagle SAH; MRI before SAH and at 1 h, 1 week, and 2 weeks	Cisterna magna Gd-DTPA with serial T1 MRI; skull-base/perineural outflow	SAH caused severe early mLV outflow failure with partial recovery by 2 weeks; CSF drainage improved readouts.	Preclinical; small groups; invasive intrathecal contrast protocol.
Cai et al. (2026)	Experimental mechanistic/cognitive-outcome evidence	Adult male C57BL/6J mice; 91 mice used. Key assays: mLV morphology/drainage *n* = 4–6/group; RNA-seq *n* = 3/group; VEGF-C/PI3K-AKT validation *n* = 5/group; *in vitro* LEC assays *n* = 3–6/group.	Delayed cognitive-impairment phase after SAH	mLV structure/drainage, metabolites, hippocampal function, VEGF-C treatment, and VEGFR3/PI3K-AKT signaling analyses	mLV fragmentation/atrophy impaired drainage and cognition; VEGF-C preserved mLV function and reduced impairment.	Preclinical; not an MRI-biomarker study; timing and intervention translation remain uncertain.

This table summarizes studies exploring the role of meningeal lymphatic vessels in stroke pathology, including immune regulation, drainage function, imaging findings, and therapeutic interventions.

AAV, adeno-associated virus; AQP4, aquaporin-4; BBB, blood–brain barrier; CCN1, cellular communication network factor 1; CD47, cluster of differentiation 47; CFSE, 5-(and 6)-carboxyfluorescein diacetate succinimidyl ester; CLN, cervical lymph node; CSF, cerebrospinal fluid; DANTE, delay alternating with nutation for tailored excitation; dCLN, deep cervical lymph node; DCE-MRI, dynamic contrast-enhanced MRI; DTI-ALPS, diffusion tensor image analysis along the perivascular space; FACS, fluorescence-activated cell sorting; FW-WM, free water in white matter; GBCA, gadolinium-based contrast agent; Gd, gadolinium; Gd-DTPA, gadopentetate dimeglumine; GFP, green fluorescent protein; ICH, intracerebral hemorrhage; IHC, immunohistochemistry; ILN, inguinal lymph node; ISF, interstitial fluid; LEC, lymphatic endothelial cell; MCAO, middle cerebral artery occlusion; MMD, moyamoya disease; mLV, meningeal lymphatic vessel; MRI, magnetic resonance imaging; PGF, placental growth factor; PI3K-AKT, phosphoinositide 3-kinase–protein kinase B pathway; PT, photothrombosis; PVSVF-WM, perivascular space volume fraction in white matter; RBC, red blood cell; RNA-seq, RNA sequencing; SAH, subarachnoid hemorrhage; scRNA-seq, single-cell RNA sequencing; snRNA-seq, single-nucleus RNA sequencing; T1-SPACE, T1-weighted sampling perfection with application-optimized contrasts using different flip-angle evolutions; THBS1, thrombospondin 1; tMCAO, transient middle cerebral artery occlusion; TTC, 2,3,5-triphenyltetrazolium chloride; VEGF-C, vascular endothelial growth factor C; VEGFR3, vascular endothelial growth factor receptor 3; VW-MRI, vessel-wall MRI; YFP, yellow fluorescent protein.

In ischemic stroke, dedicated MRI evidence for meningeal lymphatic dysfunction is still exploratory. A prospective delayed vessel-wall MRI study found ipsilateral delayed meningeal enhancement in all five patients with large cerebral infarctions, whereas none of the six patients with small infarctions showed this pattern, suggesting altered brain clearance pathways rather than collateral vascular flow (Umehana et al., 2026). Preclinical MRI studies have also used gadolinium-enhanced imaging as a treatment-response readout: VEGF-C-induced dural lymphatic growth increased CSF-ISF drainage signals and enhanced outflow via the cribriform region in mice (Keuters et al., 2025). However, these MRI findings are intervention-linked and do not yet define a standardized spontaneous imaging phenotype of post-ischemic meningeal lymphatic dysfunction.

In ICH, MRI evidence specifically targeting meningeal lymphatic dysfunction remains sparse. The best-established ICH studies demonstrate meningeal-lymphatic involvement mainly through experimental drainage assays, histological analysis, and functional manipulation rather than through dedicated MRI biomarkers (Tsai et al., 2022). Accordingly, ICH currently represents an important imaging gap in the meningeal lymphatic literature.

SAH currently provides the strongest stroke-specific MRI evidence for meningeal lymphatic dysfunction. In a beagle model of SAH, serial Gd-DTPA-enhanced T1-weighted MRI demonstrated delayed and reduced efflux along skull-base and perineural drainage routes, including the olfactory, optic, facial, vestibulocochlear, and spinal nerve regions, with only partial recovery by two weeks; intermittent cisterna magna CSF drainage was associated with improved MRI-based drainage readouts (Wang et al., 2024). Human evidence has also emerged. In patients with spontaneous SAH, multimodal MRI demonstrated reduced meningeal lymphatic vessel and deep cervical lymph node signals, which were used as surrogate readouts of meningeal lymphatic drainage. These abnormalities were more pronounced in patients who developed chronic shunt-dependent hydrocephalus, and mLV signal loss independently predicted this complication (Lu et al., 2026).

Current MRI readouts of the meningeal lymphatic system remain indirect. They primarily reflect delayed contrast enhancement or signal changes in parasagittal dural spaces, skull-base or perineural pathways, meningeal lymphatic vessel signal, or deep cervical lymph node signal, rather than direct measurements of lymphatic flow or clearance rate. Interpretation is also vulnerable to confounding by infarct size, BBB disruption, subarachnoid blood burden, timing after contrast administration, and manual region-of-interest placement. In the parasagittal DCE-MRI feasibility study, manual region-of-interest placement on a small structure was explicitly recognized as a possible reproducibility issue, and test-retest reliability was not assessed. Therefore, MRI findings on post-stroke meningeal lymphatics should currently be framed as mechanistic and early translational evidence rather than clinically validated biomarkers.

### 5.4 Summary of imaging evidence, current clinical relevance, and limitations

Taken together, available MRI evidence supports post-stroke disturbance of glymphatic pathways and, in selected settings, meningeal lymphatic pathways, but the biological directness of available markers varies markedly across modalities. The most direct observations come from experimental tracer-based MRI and selected intrathecal contrast-enhanced human studies, whereas most human stroke data still rely on indirect surrogates such as DTI-ALPS, MRI-visible PVS burden, delayed meningeal enhancement, or meningeal lymphatic vessel/deep cervical lymph node signal changes. Accordingly, these approaches should currently be regarded primarily as mechanistic and early translational tools rather than validated clinical biomarkers for routine stroke care. Their main limitations are the predominance of preclinical or invasive direct imaging, the indirect nature and methodological sensitivity of common human markers, and the limited standardization of meningeal lymphatic MRI. These constraints motivate the future research priorities outlined in the next section.

## 6 Future research directions

### 6.1 Bridging knowledge gaps in stroke-induced clearance dysfunction

Despite substantial progress, research on post-stroke glymphatic and meningeal lymphatic dysfunction remains limited by indirect biomarkers, heterogeneous stroke models, and sparse longitudinal human data (Klostranec et al., 2021; Botta et al., 2025; Machado et al., 2025; Perla et al., 2023). Future work should therefore prioritize mechanism-linked validation, subtype-specific study design, and cautious translational testing rather than assuming that clearance-targeted strategies are ready for clinical use.

A central unresolved issue is how glymphatic and meningeal lymphatic pathways interact after stroke. Most studies still assess these systems separately, even though available evidence suggests functional coupling among perivascular exchange, dural lymphatic drainage, and systemic immune responses (Esposito et al., 2019; Licastro et al., 2024; Shlobin et al., 2024). Future studies should therefore evaluate both pathways in parallel and interpret them in relation to edema evolution, BBB integrity, immune trafficking, and neurological outcome.

Another major gap concerns timing and stroke subtype. Existing longitudinal evidence from ischemic stroke and SAH suggests that clearance abnormalities may evolve from hyperacute disturbance to partial recovery or secondary degeneration (Wang et al., 2024; Pu et al., 2019; Warren et al., 2023; Eide et al., 2025), whereas comparable longitudinal data remain particularly sparse in ICH. Prospective longitudinal studies with predefined time windows are needed to determine when dysfunction peaks, whether recovery is biphasic, and which abnormalities are transient vs. persistent.

Mechanistically, future work should move beyond broad statements about AQP4 mislocalization or lymphangiogenesis and instead define stage-specific molecular and cellular drivers. Priority targets include VEGF-C/VEGFR3 signaling (Boisserand et al., 2024; Keuters et al., 2025), AQP4 anchoring and polarization pathways (Filchenko et al., 2020; Sun et al., 2022; Yang J. et al., 2024), and emerging compartmental structures such as the subarachnoid lymphatic-like membrane (Shlobin et al., 2024). Integrating single-cell or spatial omics with imaging and fluid biomarkers may help link these molecular changes to specific clearance phenotypes and outcome trajectories (Wang et al., 2023; Zhu et al., 2025).

### 6.2 Expanding imaging modalities and experimental models

Methodologically, the field now needs greater standardization and cross-validation. Different MRI- and tracer-based markers sample different compartments and do not carry the same biological meaning. Future studies should therefore pair indirect metrics such as DTI-ALPS or PVS burden with tracer-based, histological, CSF, or molecular endpoints whenever feasible. The expanding literature on noninvasive MRI methods also underscores the need to compare surrogate markers against biologically more direct reference standards.

Complementary multimodal approaches are also likely to be valuable. PET-based approaches can provide tracer-based information on CSF drainage, and multimodal PET/MRI may help integrate structural and molecular readouts (Sarker et al., 2023; Lee et al., 2020). Optical methods, including meningeal lymphatic circuit mapping, live two-photon imaging, and near-infrared fluorescence imaging, offer high spatiotemporal resolution in experimental contexts, yet their application remains largely limited to preclinical studies (Jacob et al., 2022; Wu et al., 2023; Li et al., 2024). Ultrasound- and photoacoustic-based techniques may further expand real-time assessment of CSF movement, vascular dynamics, and hemodynamics, but stroke-specific validation remains limited (Choi et al., 2024; Chen H. et al., 2024).

Model selection also requires improvement. Rodent models remain indispensable, but larger animals better approximate human CSF spaces and drainage routes. Microfluidic blood- and lymphatic-vasculature systems, together with computational models, can isolate mechanisms and generate testable predictions (Hall et al., 2024; Martinac and Bilston, 2020; Quirk et al., 2024). These platforms should be used in a coordinated rather than interchangeable manner.

Finally, therapeutic translation should remain explicitly cautious. Modulation of meningeal lymphatic drainage, VEGF-C signaling, or AQP4-dependent clearance remains mechanistically promising but largely preclinical (Boisserand et al., 2024; Keuters et al., 2025; Yang J. et al., 2024). Recent work also suggests that lymphatic responses may be phase-dependent rather than uniformly beneficial (Boisserand et al., 2024). Early translational studies should therefore prioritize safety, timing, biological target engagement, and subtype-specific endpoints rather than assuming efficacy. Together, these priorities may help clarify whether post-stroke glymphatic and meningeal lymphatic abnormalities are useful biomarkers, causal therapeutic targets, or both, and under which stroke subtypes and time windows translation is justified.

### 6.3 Limitations of current evidence and of this review

This review has several limitations. First, the available evidence is dominated by animal models and relatively small observational human studies, with few prospective longitudinal datasets and very limited biomarker validation in interventional settings. Second, ischemic stroke, ICH, and SAH differ substantially in edema evolution, blood-product toxicity, intracranial pressure, BBB disruption, and immune trafficking, which limits direct comparison and broad pooling of mechanisms and imaging findings across stroke subtypes. Third, many of the imaging markers discussed here are indirect. DTI-ALPS does not directly measure bulk flow or clearance rate and is sensitive to white-matter geometry and acquisition parameters. MRI-visible perivascular spaces are biologically nonspecific. DCE-MRI, although more closely related to tracer transport, has limited routine clinical feasibility when contrast administration or intrathecal protocols are required. Fourth, biological and technical confounders, including sleep state, edema, white-matter injury, vascular pulsatility, time from stroke onset, scanner platform, and protocol variation, complicate interpretation and reduce comparability across studies. Finally, this article should be interpreted as a narrative review with a structured literature search rather than as a formal quantitative assessment of certainty or clinical readiness.

## 7 Conclusion

In summary, stroke may disrupt brain fluid and waste clearance pathways, including the glymphatic and meningeal lymphatic systems, across ischemic stroke, ICH, and SAH. Experimental studies and early human imaging suggest abnormalities in CSF dynamics, BBB integrity, and clearance-related MRI markers, but the strength and directness of the evidence vary substantially across stroke subtypes and imaging modalities. At present, human evidence remains limited and is still dominated by indirect markers, particularly DTI-ALPS and MRI-visible perivascular spaces. These approaches should therefore be regarded primarily as mechanistic and investigational tools rather than validated biomarkers for routine diagnosis or treatment guidance. Therapeutic strategies that target clearance pathways, including VEGF-C-related lymphangiogenesis, AQP4-related mechanisms, and CSF drainage, remain promising but experimental, and their optimal timing, safety, and translational efficacy remain uncertain. Future subtype-specific, longitudinal, multimodal, and prospectively validated human studies are needed to determine whether monitoring or modulating brain clearance pathways can improve functional recovery and long-term cognitive outcomes after stroke.
