# Supplementary material for: Post-stroke glymphatic and meningeal lymphatic dysfunction: mechanisms, imaging evidence, and translational challenges
Source: Front Stroke. 2026 Jul 31;5:1861562. doi: 10.3389/fstro.2026.1861562 (PMC13472805; doi:10.3389/fstro.2026.1861562)
Supplement: Supplementary file 1 [file Table_1.docx]

**Supplementary critical evidence tables**

# **Supplementary Table S1. Critical evidence summary of studies investigating post-stroke glymphatic dysfunction**

| **Study/ref.** | **Evidence category/directness** | **Population and sample size** | **Stroke subtype/model and timing** | **Imaging/protocol/readout** | **Outcome definition** | **Reported effect size/result and direction** | **Evidence quality/readiness** | **Key limitations/confounders** |
| --- | --- | --- | --- | --- | --- | --- | --- | --- |
| **Ischemic stroke / cerebral infarction - experimental and mixed translational evidence** | | | | | | | | |
| **Mestre et al. [30]** | Experimental direct physiological imaging/tracer evidence. | Male C57BL/6, Glt1-GCaMP7, NG2-dsRed, and *Aqp4*^-/-^ mice in multimodal focal-ischemia/MCAO experiments; no single total n reported. Key assays: macroscopic CSF tracer/rCBF imaging n = 5; cortical water content n = 4–7 mice/time point; intravenous tracer uptake n = 7; CSF tracer uptake n = 6; 3D-FIESTA cisternography n = 7 sham and n = 8 MCAO; GCaMP/CSF front n = 6; 2P SI/CSF experiments n = 4; Aqp4 genotype assays n = 4–6/group. | Acute ischemic stroke; hyperacute minutes-to-hours after arterial occlusion and spreading depolarization. | MRI, radiolabeled tracers, and multiphoton imaging of perivascular CSF influx. | CSF influx into ischemic tissue, PVS enlargement, and acute tissue swelling. | CSF rapidly entered ischemic tissue; spreading depolarizations and vasoconstriction enlarged PVS and accelerated CSF influx. Direction: increased influx/edema, not increased effective clearance. | Direct preclinical physiology; strong mechanistic directness for hyperacute CSF influx; low clinical readiness. | Rodent hyperacute physiology; mainly edema-driving influx biology; not a validated clinical clearance biomarker. |
| **Gaberel et al. [57]** | Experimental direct tracer-based MRI. | Mouse experimental stroke models; key MRI quantification n = 5/group; assay-specific n varied. | Experimental stroke models; early post-stroke imaging including acute 3- and 24-h windows. | Contrast-enhanced MRI after CSF-space gadolinium administration to assess brain parenchymal tracer enhancement/glymphatic perfusion. | Glymphatic perfusion after stroke and response to fibrinolysis in the experimental setting. | SAH and acute embolic ischemic stroke markedly impaired glymphatic perfusion, whereas carotid ligation and ICH did not. Intracerebroventricular tissue-type plasminogen activator improved perfusion after SAH, and spontaneous arterial recanalization restored glymphatic function after embolic ischemic stroke. Direction: model- and recanalization-dependent changes in glymphatic perfusion. | Direct preclinical tracer MRI; useful mechanistic evidence; low clinical biomarker readiness. | Small preclinical groups; mixed experimental stroke contexts; not a standardized clinical MRI biomarker. |
| **Zhang J et al. [58]** | Experimental direct tracer-based MRI. | Male C57BL/6 mice; MRI n = 3/group; pathology/AQP4 analysis n = 4/group. | Permanent MCAO with intact BBB; MRI began about 28 min after occlusion and 14 min after BOPTA-Gd injection. | Intracisternal BOPTA-Gd (~4 µL, 0.6 µL/min for 6 min); 11.7-T T1WI tracer kinetics with DWI/ADC and AQP4 polarization assessment. | Tracer perfusion kinetics in cortex/striatum/ventricles; ADC and AQP4 polarization. | Ipsilateral cortex/striatum and bilateral ventricular tracer perfusion were reduced/delayed; T1WI signal correlated positively with ADC. Direction: acute ischemic edema associated with suppressed glymphatic transport and AQP4 depolarization. | Direct high-field preclinical tracer MRI; biological directness high, but sample very small. | Very small MRI sample; hyperacute permanent-occlusion model; high-field protocol not equivalent to routine human MRI. |
| **Warren et al. [80]** | Experimental fluorescent CSF-tracer transport assay. | Photothrombotic stroke mice. Brain tissue: 24 h sham n = 6, stroke n = 8; 2 weeks sham n = 8, stroke n = 12. Nasal mucosa: 24 h sham n = 6, stroke n = 6; 2 weeks sham n = 4, stroke n = 6. | Photothrombotic ischemic stroke; 24 h and 2 weeks after stroke. | Fluorescent CSF tracer entry into brain parenchyma and tracer outflow to nasal mucosa/cribriform-plate route. | CSF tracer influx into brain and efflux to nasal mucosa. | CSF tracer influx and nasal outflow were reduced at 24 h but not at 2 weeks. Direction: early reduction in CSF circulation/outflow, with no detectable stroke–sham difference at 2 weeks. | Direct preclinical tracer assay; not MRI; useful for timing biology. | Animal-only endpoint; attrition from death/failed stroke; not directly translatable to clinical clearance measurement. |
| **Lin et al. [81]** | Experimental direct tracer-based MRI. | 12 Sprague–Dawley rats: sham/control n = 4, acute MCAO 1 day n = 4, subacute MCAO 7 days n = 4. | Left MCAO; imaging at 1 day or 7 days after operation. | Intrathecal Gd-DTPA (2 µL/min; total 20 µL) followed by high-resolution 3D T1-weighted contrast-enhanced MRI for 6 h; regional analysis in substantia nigra and ventral thalamic nucleus. | Contrast kinetics in secondary-degeneration regions remote from infarct core. | Secondary-degeneration regions showed abnormal tracer kinetics, including delayed/retained enhancement in ipsilateral substantia nigra and ventral thalamic nucleus. Direction: regionally impaired glymphatic transport in ipsilateral remote degeneration areas. | Direct preclinical tracer MRI; region-specific mechanistic evidence. | Very small n = 4/group; remote degeneration rather than infarct-core readout; no human validation. |
| **Li C et al. [82]** | Experimental longitudinal direct tracer-based MRI plus pathology. | 20 adult male Sprague–Dawley rats: sham n = 5, MCAO 1 week n = 5, MCAO 2 weeks n = 5, MCAO 2 months n = 5. | Transient MCAO with 90-min occlusion/reperfusion; DCE-MRI and pathology at 1 week, 2 weeks, and 2 months after MCAO. | 3.0-T intrathecal Gd-DTPA DCE-MRI; neurological scoring; GFAP, AQP4, Iba1, and APP immunofluorescence in thalamic ventroposterior nucleus. | Thalamic tracer retention/kinetics, AQP4 polarization, glial activation, and APP deposition. | Astrocyte/microglial activation and APP deposition peaked at 2 weeks; AQP4 polarization was lowest at 2 weeks and 2 months. Direction: thalamic glymphatic impairment most evident around 2 weeks after MCAO. | Longitudinal preclinical tracer MRI with pathology; stronger temporal information than single-time-point models. | Small longitudinal groups; secondary-region thalamic readout; incomplete correlation between all MRI metrics and histology. |
| **Zhu J et al. [42]** | Mixed translational evidence: animal mechanism plus human indirect MRI. | Experimental mouse MCAO models plus 18 adult patients with AIS after endovascular treatment; all 18 underwent DTI at 24 ± 4 h, 10 underwent repeat DTI at 72 ± 12 h, and 5 underwent a third DTI at day 7 ± 1. | Animal tMCAO during the first week; human DTI at 24 ± 4 h, 72 ± 12 h, and day 7 ± 1 after endovascular treatment. | Animal ex vivo fluorescence imaging, in vivo two-photon imaging, MRI, and glymphatic modulation; human DTI-ALPS with edema/outcome assessment. | Edema evolution, glymphatic readouts, and exploratory human edema/outcome associations. | Glymphatic dysfunction tracked edema evolution after MCAO; enhancing glymphatic function reduced day-2 edema, whereas suppression delayed day-7 edema absorption. Human AIS showed lower ALPS related to edema severity/outcome. Direction: impaired/lower readout associated with edema and worse outcome. | Translational bridge but human evidence remains exploratory and indirect. | Small human component; DTI-ALPS may be confounded by edema, reperfusion injury, and white-matter changes. |
| **Ischemic stroke / cerebral infarction - human indirect MRI or exploratory imaging evidence** | | | | | | | | |
| **Qin et al. [62]** | Human indirect MRI surrogate. | 20 analyzed subacute ischemic stroke patients with single left subcortical lesions and 30 healthy controls; 6/26 patients and 2/32 controls excluded before analysis. | Subacute ischemic stroke; DTI 7–40 days after onset, median 9 days. | DTI-ALPS with bilateral ROI analysis; Fugl–Meyer motor and sensory scores and corticospinal-tract DTI metrics. | ALPS index and motor dysfunction by Fugl–Meyer motor score. | Left ALPS was lower in stroke patients than controls (1.424 ± 0.132 vs 1.565 ± 0.197; P = 0.004); higher left ALPS correlated with better simplified Fugl–Meyer motor score (ρ = 0.52, P = 0.019). Direction: lower ALPS associated with worse motor dysfunction. | Human indirect regional surrogate; exploratory association, not flow validation. | Small left-lesion-only cohort; lesion-involving ROIs excluded; no longitudinal or tracer-based validation. |
| **Toh and Siow [83]** | Human indirect MRI surrogate. | 50 ischemic stroke patients and 44 healthy controls; 32 right-sided and 18 left-sided infarcts; 19/50 had hemorrhagic change. | Ischemic stroke; onset-to-MRI mean 17.1 ± 14.8 days, range 1–60 days. | DTI-ALPS with bilateral ALPS index and infarct/contralateral comparison; ROI placement avoided infarcted tissue. | Ipsilateral versus contralateral ALPS, infarct volume, and time since onset. | Ipsilateral ALPS was lower than contralateral ALPS (1.162 ± 0.126 vs 1.335 ± 0.160, P < 0.001); ALPS was lower with larger infarct volume and within ≤14 days, and was higher at longer onset-to-MRI intervals. Direction: lower ipsilesional ALPS, with higher values at longer onset-to-MRI intervals. | Human cross-sectional indirect surrogate; clinically feasible but biologically indirect. | Single-center cross-sectional study; variable timing and lesion size; hemorrhagic transformation and white-matter geometry may confound ALPS. |
| **Zeng et al. [65]** | Human prospective indirect MRI surrogate with short follow-up. | 59 AIS patients and 29 age-/sex-matched healthy controls; 18 patients underwent follow-up MRI. | AIS; baseline onset-to-MRI 5.9 ± 3.6 days; follow-up MRI at mean 62 days. | DTI-ALPS; ipsilateral/contralateral ALPS comparison; FMA-UE, NIHSS, infarct volume, and follow-up ALPS change. | Upper-limb motor deficit, neurological severity, and longitudinal ALPS change. | Ipsilateral ALPS was lower than contralateral ALPS (1.225 ± 0.132 vs 1.337 ± 0.138, P < 0.001); ipsilateral ALPS correlated positively with FMA-UE (ρ = 0.404, P = 0.001) and negatively with NIHSS (ρ = −0.484, P < 0.001); ALPS increased at follow-up (1.296 ± 0.141 vs 1.174 ± 0.109, P = 0.001). Direction: lower ALPS associated with worse motor/neurological deficit and partial recovery. | Prospective human indirect surrogate; limited longitudinal support. | Single-center, relatively small cohort; only 18 follow-up scans; mainly mild AIS; ALPS does not measure whole-brain glymphatic flow. |
| **Wang Y et al. [66]** | Human prospective indirect MRI surrogate. | 29 acute subcortical infarct patients and 25 healthy controls after exclusions; initially screened 42 stroke patients and 26 controls. | Acute subcortical infarction within 72 h; onset time median 36 h (range 6–70 h); MoCA at 7 and 90 days. | 3.0-T MRI with DTI/structural sequences; bilateral DTI-ALPS; MoCA; ROC analysis for early PSCI. | Early post-stroke cognitive impairment and 7-day MoCA. | Lesion-side and non-lesion-side ALPS were lower than controls (1.371 ± 0.170 and 1.310 ± 0.198 vs 1.568 ± 0.115; both P < 0.001); lesion-side ALPS correlated with 7-day MoCA (r = 0.510, P = 0.005); AUC for cognitive impairment = 0.868. Direction: lower ALPS associated with poorer cognition. | Prospective human indirect biomarker study; predictive signal requires validation. | Small single-center cohort; restricted to small subcortical infarcts without thrombolysis/thrombectomy; specificity limited. |
| **Zhu Z et al. [84]** | Human acute-phase indirect MRI surrogate. | 103 participants: 69 AIS patients (35 left-sided, 34 right-sided infarctions) and 34 healthy controls. | First-ever unilateral AIS in basal ganglia and/or corona radiata; MRI within 72 h of onset. | 3.0-T MRI; 30-direction DTI; automated ROI DTI-ALPS; NIHSS and FMA-UE with hemispheric laterality analysis. | ALPS lateralization, early neurological severity, and upper-limb motor function. | Left-hemisphere ALPS was lower in both left- and right-sided infarct groups than in HCs (HCs 2.547 ± 0.600 vs left-sided infarction 2.115 ± 0.548, P = 0.0069; HCs vs right-sided infarction 2.193 ± 0.587, P = 0.035). Left ALPS correlated with NIHSS and FMA-UE in left-sided infarction and with FMA-UE in right-sided infarction. Direction: lower left ALPS was associated with worse early motor/neurological status. | Human acute indirect surrogate; automated ROI improves consistency but clinical validation remains early. | Cross-sectional, single-center, acute-only study; no infarct-volume or etiologic stratification; biological basis of lateralization uncertain. |
| **Finkelstein et al. [85]** | Human post-treatment indirect MRI surrogate. | 55 analyzable anterior-circulation LVO patients after thrombectomy; larger screened thrombectomy cohort reported but final analysis used n = 55. | AIS after thrombectomy; early post-treatment MRI, most often approximately 24 h; 90-day functional outcome. | Post-thrombectomy DTI-ALPS–derived glymphatic metric; infarcted versus contralateral side; futile recanalization defined by poor 90-day mRS despite successful reperfusion. | Futile recanalization/90-day mRS and baseline NIHSS association. | The infarct-side DTI-ALPS index was lower than the contralateral index (P = 0.035); patients with futile recanalization had a lower DTI-ALPS index than functionally independent patients (P = 0.049); the index was inversely associated with presenting NIHSS (ρ = −0.46, P = 0.002). Direction: a lower DTI-ALPS index was associated with worse outcome. | Human post-treatment observational study; clinically relevant outcome but indirect MRI construct. | Highly selected thrombectomy cohort; exclusions due ROI involvement/mass effect; observational association does not prove causality. |
| **Umehana et al. [89]** | Human exploratory meningeal-lymphatic/delayed enhancement MRI evidence included in Table 1 as clearance-related imaging. | 11 patients with recent cerebral infarction; 6 patients with moyamoya disease as comparators. | Subacute cerebral infarction; MRI performed 11.5 ± 4.5 days after onset; delayed imaging 1–6 h after GBCA administration. | Whole-brain DANTE T1-SPACE vessel-wall MRI acquired before, immediately after, and 1–6 h after GBCA administration. | Ipsilateral delayed meningeal enhancement in relation to infarct size. | All 5 large infarctions showed ipsilateral delayed meningeal enhancement; 0/6 small infarctions did. Direction: large infarction associated with delayed ipsilateral enhancement. | Human exploratory indirect clearance-pathway imaging; not a direct flow measure. | Very small sample; delayed enhancement may be confounded by infarct size, BBB disruption, vascular enhancement, and contrast timing. |
| **Intracerebral hemorrhage - experimental and human evidence** | | | | | | | | |
| **Liu et al. [34]** | Experimental mechanistic intervention evidence. | 75 male Sprague–Dawley rats: sham n = 25, ICH n = 25, ICH + cerebral/cervical lymphatic blocking n = 25; assay-specific n commonly varied by readout. | Collagenase-induced rat ICH; lymphatic blocking performed before ICH; readouts mainly at 3 and 7 days after ICH. | Cervical lymphatic-node removal/ligation; collagenase IV ICH model; Garcia score, brain water content, hematoma volume, cytokines, AQP4, GFAP, active caspase-3. | Neurological deficit, perihematomal edema, inflammation, AQP4/GFAP expression, and apoptosis. | Drainage blockade aggravated neurological deficit, edema, inflammatory imbalance, astrocytic activation, and neuronal apoptosis. Direction: impaired drainage worsened ICH injury. | Preclinical causal/intervention evidence; mechanism relevant but not imaging validation. | Animal-only; blockade may have off-target immune/drainage effects; not a human imaging biomarker study. |
| **Zhang C et al. [63]** | Human indirect MRI surrogate. | 20 left-hemisphere spontaneous ICH patients and 31 healthy controls after exclusions; initially screened 23 ICH patients and 33 controls. | Subacute/chronic mixed ICH; disease duration 21–155 days; MRI approximately 1 month after conservative treatment. | DTI and SWI; DTI-ALPS index measured on lesion and nonlesion sides. | Lesion-side versus nonlesion/healthy-control ALPS and association with disease duration. | Lesion-side ALPS was lower than the contralateral side and controls; lesion-side ALPS correlated positively with disease duration (r = 0.537, P = 0.018). Direction: lower lesion-side ALPS, with higher values at longer disease durations. | Human indirect surrogate; small exploratory dataset. | Small sample; only left-hemisphere ICH; manual ROI placement; no functional outcome analysis; perihematomal distortion may confound diffusion metrics. |
| **Xia et al. [16]** | Human exploratory indirect MRI surrogate. | 58 mild-to-moderate spontaneous ICH patients and 63 age-/sex-matched healthy controls; acute-subacute n = 22, chronic n = 36. | Retrospective ICH cohort; CT and MRI interval ≤24 h; subgroups by disease duration ≤3 weeks versus >3 weeks; 90-day functional outcome assessed. | DTI-ALPS plus CT-based hematoma/perihematomal edema quantification, MoCA, mediation/logistic regression, and ROC analysis. | Hematoma volume, MoCA, 90-day poor outcome. | ALPS was lower in ICH than controls (FDR-adjusted P < 0.001). Acute-subacute ALPS correlated negatively with hematoma volume (r = −0.572, FDR-adjusted P = 0.031); chronic ALPS correlated positively with MoCA (r = 0.425, FDR-adjusted P = 0.014). ALPS independently predicted poor 90-day outcome (β = −9.851, P = 0.018; AUC = 0.776). Direction: lower ALPS associated with larger hematoma, worse cognition, and poorer prognosis. | Human retrospective exploratory biomarker study; includes outcome modeling but no external validation. | Single-center; mild-to-moderate ICH only; DTI-ALPS affected by hematoma, edema, lesion location, and white-matter distortion. |
| **Cai J et al. [86]** | Human acute ICH indirect MRI surrogate. | 55 acute spontaneous ICH patients, including 46 supratentorial and 9 infratentorial/subtentorial cases, plus 97 age-/sex-matched controls. | Acute spontaneous ICH; 90-day mRS outcome categorized as favorable (mRS ≤2) versus poor (mRS >2). | Conventional MRI plus DTI; DTI-ALPS; hemorrhage volume on SWI, edema volume on T2-weighted imaging, relative edema ratio; ipsilateral/contralateral ALPS for supratentorial cases. | Ipsilateral ALPS, hemorrhage/edema burden, and 90-day functional outcome. | Ipsilateral ALPS was lower than controls (1.34 ± 0.24 vs 1.46 ± 0.22, P = 0.003); higher ipsilateral ALPS was independently associated with favorable 90-day outcome (OR = 1.686 per 0.1 increase, P = 0.038). Direction: lower ALPS associated with greater blood/edema burden and worse outcome. | Human acute observational biomarker study; clinically relevant outcome but indirect MRI metric. | Observational acute cohort; DTI-ALPS is not tracer-based; ipsilateral analysis mainly applies to supratentorial ICH; hemorrhage/edema may confound diffusion metrics. |
| **Subarachnoid hemorrhage - experimental and human evidence** | | | | | | | | |
| **Goulay et al. [35]** | Experimental large-animal direct CSF-circulation evidence. | 3 adult cynomolgus monkeys in a nonhuman primate SAH model. | Physiological baseline and acute post-SAH assessment. | MRI tracking of temporal DOTA-Gd distribution after CSF injection to assess brain parenchymal CSF circulation. | Brain parenchymal CSF tracer circulation before versus after SAH. | CSF entered the brain parenchyma under physiological conditions, whereas experimental SAH severely impaired parenchymal CSF circulation. Direction: marked reduction after SAH. | Direct large-animal tracer MRI; high biological directness but very small sample. | n = 3; invasive tracer protocol; acute experimental model; no clinical biomarker threshold. |
| **Wang J et al. [36]** | Experimental large-animal direct tracer-based MRI. | 14 male beagles aged 10–12 months: sham n = 3, SAH day 1 n = 3, SAH week 2 n = 4, SAH + CSF drain n = 4; some MRI quantifications used n = 3 or 6. | Beagle SAH by endovascular perforation; MRI before SAH and at 1 h, 1 week, and 2 weeks; cisterna magna CSF drainage from day 1 to day 3 in treatment group. | Cisterna magna Gd-DTPA injection with serial 3D T1-weighted MRI; glymphatic influx/efflux, PVS, ventricles, skull-base/perineural outflow, cervical lymph node signal, and CSF drainage response. | Glymphatic and meningeal lymphatic signal intensity/time course; hydrocephalus and neurological/cognitive readouts. | SAH caused severe early impairment of glymphatic influx/efflux and delayed/reduced meningeal lymphatic outflow, persisting through 1 week with partial recovery by 2 weeks; intermittent CSF drainage improved clearance-related readouts. Direction: severe early dysfunction with partial recovery. | Direct preclinical large-animal tracer MRI; strong compartmental readout, low routine clinical readiness. | Small groups; anesthesia may affect CSF/glymphatic dynamics; invasive intrathecal contrast protocol; not routine clinical imaging. |
| **Pu et al. [37]** | Experimental biological support; non-MRI. | Three-month-old male C57BL/6 mice; sham and SAH groups; tracer/histology/T-cell assays commonly n = 4/group; western blot/cytokine/apoptosis assays n = 4–5/group; behavioral assays n = 8/group. | Mouse SAH induced by 60 µL autologous venous blood into cisterna magna; analyses mainly at 1 day and 1 week; behavior at 7 days. | Intracisternal Texas Red-dextran tracer; meningeal lymphatic and dCLN tracer distribution; AQP4 polarization, tau, T cells, glial activation, cytokines, apoptosis, Garcia score, open-field behavior. | Brain tracer penetration, meningeal/dCLN drainage, inflammation/apoptosis, and behavior. | SAH impaired meningeal lymphatic drainage to dCLNs and reduced brain parenchymal tracer penetration, with tracer accumulation in mLVs, AQP4 depolarization, tau/T-cell accumulation, neuroinflammation, apoptosis, and behavioral deficits. Direction: persistent drainage malfunction after SAH. | Mechanistic preclinical tracer/histology support; not MRI. | Mouse cisterna-magna blood-injection model; small assay-specific n; supports mechanism but not human imaging evidence. |
| **Eide et al. [87]** | Human direct tracer-based MRI. | 27 SAH patients and 22 age-/sex-matched reference subjects without neurological or CSF disease; SAH subgroups: <3 months n = 5, 3–6 months n = 10, 6–12 months n = 5, >12 months n = 7. | Human post-SAH time-evolution MRI from early subacute to chronic periods. | Intrathecal gadobutrol-enhanced glymphatic MRI using 0.50 mmol gadobutrol as CSF tracer; standardized multiphase T1-weighted MRI; tracer enrichment in brain, subarachnoid spaces, ventricular CSF, and perivascular subarachnoid spaces. | Brain tracer enrichment and redistribution from subarachnoid spaces toward ventricles over time after SAH. | SAH patients showed marked impairment of glymphatic enrichment, especially cortex and subcortical white matter after 24 h, with CSF tracer redistribution toward ventricles; alterations were most pronounced at 3–6 months and less after 12 months. Direction: severe human glymphatic impairment after SAH. | Most direct human MRI evidence in this table; still invasive and based on small subgroups. | Intrathecal contrast protocol; small subgroup sizes; heterogeneous time from bleed; not routine clinical imaging. |
| **Oka et al. [17]** | Human indirect MRI surrogate. | 60 aneurysmal SAH patients. | DTI at median 18 days after onset; 3-month mRS outcome and shunt-dependent hydrocephalus assessed. | DTI-ALPS index from deep white-matter directional diffusivity; ALPS <1 analysis; mediation analysis for hydrocephalus between low ALPS and poor outcome. | Poor 3-month functional outcome and shunt-dependent hydrocephalus. | Poor-outcome patients had lower ALPS than favorable-outcome patients (1.09 [0.91–1.17] vs 1.29 [1.16–1.44], P = 0.0002). Low ALPS had total effect on poor outcome (OR = 7.98, 95% CI 1.12–56.79), partly mediated by hydrocephalus (indirect-effect OR = 3.11, 95% CI 1.23–7.89). Direction: lower ALPS associated with poor outcome and hydrocephalus. | Human indirect biomarker with outcome association; no tracer validation. | Single-center retrospective study; subacute single-time-point MRI; mediation analysis does not prove causality. |
| **Lu et al. [90]** | Human exploratory multimodal MRI surrogate. | 58 acute spontaneous SAH patients, 26 patients with post-SAH chronic shunt-dependent hydrocephalus, and 41 healthy controls. Glymphatic MRI metrics were available in 57 SAH patients and 41 controls; mLV/dCLN signal MRI was available in 58 SAH patients and 41 controls; chronic-hydrocephalus sequence-level n = 22 for DWI-derived PVS metrics and n = 24 for T2-derived mLV/dCLN signal metrics. | Acute spontaneous SAH and an independent cohort with chronic post-SAH hydrocephalus. | DTI-ALPS index, FW-WM, PVSVF-WM, and signal intensity of mLVs and dCLNs. | Glymphatic and meningeal lymphatic surrogate signals and chronic shunt-dependent hydrocephalus risk. | Compared with healthy controls, SAH patients showed reduced DTI-ALPS and mLV/dCLN signal intensity, with increased FW-WM and PVSVF-WM. These abnormalities were more pronounced in chronic shunt-dependent hydrocephalus. Reduced DTI-ALPS and mLV signal loss independently predicted chronic shunt-dependent hydrocephalus. Direction: more abnormal MRI-derived glymphatic and meningeal lymphatic surrogate measures were associated with chronic hydrocephalus and poorer functional outcomes. | Human multimodal observational surrogate evidence; clinically relevant but indirect. | Single-center exploratory cohort; mLV/dCLN signal intensities are indirect drainage surrogates, not direct lymphatic-flow measurements. |

Notes: 2P = two-photon microscopy; 3D-FIESTA = three-dimensional fast imaging employing steady-state acquisition; ADC = apparent diffusion coefficient; AIS = acute ischemic stroke; ALPS = analysis along the perivascular space; APP = amyloid precursor protein; AQP4 = aquaporin-4; AUC = area under the curve; BBB = blood–brain barrier; BOPTA-Gd = gadobenate dimeglumine; CI = confidence interval; CSF = cerebrospinal fluid; CT = computed tomography; DANTE = delay alternating with nutation for tailored excitation; dCLN = deep cervical lymph node; DCE-MRI = dynamic contrast-enhanced MRI; DOTA-Gd = gadoterate meglumine; DTI = diffusion tensor imaging; DTI-ALPS = diffusion tensor image analysis along the perivascular space; DWI = diffusion-weighted imaging; FDR = false discovery rate; FMA-UE = Fugl–Meyer Assessment of the Upper Extremity; FW-WM = free water in white matter; GBCA = gadolinium-based contrast agent; Gd-DTPA = gadopentetate dimeglumine; dsRed = Discosoma red fluorescent protein; GCaMP = genetically encoded calcium indicator; GFAP = glial fibrillary acidic protein; Glt1 = glutamate transporter 1; HC = healthy control; ICH = intracerebral hemorrhage; Iba1 = ionized calcium-binding adaptor molecule 1; LVO = large-vessel occlusion; MCAO = middle cerebral artery occlusion; mLV = meningeal lymphatic vessel; MoCA = Montreal Cognitive Assessment; MRI = magnetic resonance imaging; mRS = modified Rankin Scale; NIHSS = National Institutes of Health Stroke Scale; OR = odds ratio; PSCI = post-stroke cognitive impairment; PVSVF-WM = perivascular space volume fraction in white matter; PVS = perivascular space; rCBF = relative cerebral blood flow; NG2 = neuron-glial antigen 2; ROC = receiver operating characteristic; ROI = region of interest; SAH = subarachnoid hemorrhage; SI = signal intensity; SWI = susceptibility-weighted imaging; T1-SPACE = T1-weighted sampling perfection with application-optimized contrasts using different flip-angle evolutions; T1WI = T1-weighted imaging; tMCAO = transient middle cerebral artery occlusion. DTI-ALPS, delayed enhancement, mLV signal, and dCLN signal are indirect surrogates and should not be interpreted as direct flow or clearance-rate measures.

# **Supplementary Table S2. Critical evidence summary of studies investigating post-stroke meningeal lymphatic dysfunction**

| **Study/ref.** | **Evidence category/directness** | **Population and sample size** | **Stroke subtype/model and timing** | **Imaging/protocol/readout** | **Outcome definition** | **Reported effect size/result and direction** | **Evidence quality/readiness** | **Key limitations/confounders** |
| --- | --- | --- | --- | --- | --- | --- | --- | --- |
| **Ischemic stroke / cerebral infarction - mechanistic and imaging evidence** | | | | | | | | |
| **Esposito et al. [43]** | Experimental brain-to-cervical-lymph-node signaling evidence. | Male Sprague–Dawley rats (320–340 g) and male C57BL/6 mice (24–27 g); assay-specific n. Rat CLN activation IHC n = 4/group, VEGFR3 western blot n = 3/group, rat FACS sham n = 3/MCAO n = 11/MCAO+MAZ51 n = 7. Mouse CLN qPCR/western/IHC commonly n = 3–13/group; mouse MAZ51 infarct MCAO n = 8 and MCAO+MAZ51 n = 9; superficial CLN-removal infarct control n = 10/removal n = 11; ILN-removal control n = 7/removal n = 6. | Early post-ischemic phase. | CSF/CLN VEGF-C/VEGFR3 assays, immune profiling, and CLN/VEGFR3 interventions. | Brain-to-CLN lymphatic-immune signaling and inflammatory injury. | Ischemia activated VEGF-C/VEGFR3 brain-to-CLN lymphatic-immune signaling; pathway blockade reduced inflammatory injury. Direction: ischemia increased lymphatic-immune signaling. | Mechanistic preclinical evidence; biological plausibility strong, imaging readiness low. | Not a clinical imaging biomarker; assay-specific sample sizes varied. |
| **Yanev et al. [46]** | Experimental genetic/developmental evidence (not mLV-specific). | Adult male C57BL/6 and *Prox1*-tdTomato mice, and *Vegfr3*^wt/wt^ and *Vegfr3*^wt/mut^ mice on a C3H background; PT surgeries n = 27; tMCAO surgeries n = 34 attempted; figure-level n typically PT n = 5–6 versus sham n = 4 and tMCAO n = 5–7 versus sham n = 5. | Experimental ischemic stroke induced by PT or tMCAO; acute/subacute outcomes. | PT and tMCAO in mice with developmental mLV hypoplasia caused by reduced VEGFR3 signaling; whole-mount mLV analysis and stroke-outcome assessment. | Post-stroke lesion/outcome in relation to mLV developmental status. | PT, but not tMCAO, induced mLV growth; *Vegfr3*^wt/mut^ mice had larger infarcts after tMCAO, but not after PT. Direction: effects were stroke-model dependent. | Causal preclinical evidence, but based on developmental deficiency. | Global developmental VEGFR3 impairment is not mLV-specific and may not model acute therapeutic modulation in adults. |
| **Boisserand et al. [44]** | Experimental prophylactic intervention evidence. | Male C57BL/6J and *Vegfr3*::YFP mice (4–10 weeks); no single total n across all experiments. Key assays: CSF-MRI/DCE-MRI n = 10/group (20 mice); dural LEC sorting n = 30 mice/group; brain snRNA-seq n = 5/group; tMCAO cohort n = 108 with 31 deaths and 8 MRI/hemorrhage exclusions; behavior and lesion/dCLN-cauterization analyses commonly n = 5–13/group; post-stroke VEGF-C156S assays n = 5–7/group. | Pre-stroke VEGF-C prophylaxis with early/subacute post-stroke readouts. | AAV-VEGF-C delivery; drainage to dCLNs, inflammation, and motor outcome readouts. | CSF lymphatic drainage, peri-infarct inflammation, and recovery-related behavior. | VEGF-C prophylaxis increased CSF lymphatic drainage and modulated neuroinflammation with improved recovery. Direction: enhanced drainage associated with reduced inflammation/recovery benefit. | Interventional preclinical evidence; timing-specific and not directly clinical. | Prophylactic design limits translation; timing likely critical. |
| **Keuters et al. [47]** | Experimental interventional MRI evidence. | Young adult male C57BL/6JOlaHsd mice in the VEGF-C groups and C57BL/6JRj mice in the control groups. Reported post-exclusion totals across cohorts: tMCAO + VEGF-C n = 50, sham + VEGF-C n = 35, tMCAO + control n = 49, and sham + control n = 34. qPCR cohort: n = 6/group (analyzed n = 5–6/group). Gd-enhanced MRI cohort after exclusions: n = 7/6/6/6, respectively. | tMCAO; AAV9-VEGF-C administered 14 or 35 days before stroke; Gd-enhanced dynamic MRI at 24 h and behavioral/histological outcomes at 3–7 days. | Gadolinium-enhanced MRI of CSF/ISF drainage and cribriform outflow; behavioral and histological readouts. | MRI drainage/outflow signal, astroglial reactivity, selected behavior/histology. | VEGF-C expanded dural lymphatic vessels, altered CSF/ISF tracer dynamics and enhanced cribriform outflow, improved gait and neurological scores, and shifted glial responses; infarct and edema volumes and some sensorimotor measures were unchanged. Direction: selected drainage and functional readouts improved, but structural and behavioral effects were not uniform. | Preclinical MRI with treatment-linked readout; useful mechanistic imaging but not a spontaneous human phenotype. | Prophylactic AAV dosing 14–35 days before stroke limits clinical translation; treatment was confounded with mouse substrain/vendor; intracerebroventricular Gd-MRI measured experimentally administered tracer dynamics rather than spontaneous clinical mLV flow. |
| **Choi et al. [45]** | Experimental timing-dependent mechanistic intervention evidence. | 10–12-week-old male mice. tMCAO time-course lymphatic/MRI analyses used n = 8 sham and n = 9 tMCAO at each time point. dCLN analyses n = 6/group; peripheral lymph-node controls n = 3/group; Gd CSF-MRI n = 3 sham and n = 5 tMCAO; VEGF-C/immune doublet assays n = 3/group; MAZ51 CP lymphatic and infarct analyses n = 6/group; VEGF-C156S analyses n = 3/group for lymphatics, n = 3–4/group for infarct, and n = 3–4/group for CD45. | Acute and later post-stroke phases. | VEGF-C/VEGFR3 manipulation; lymphangiogenesis, infarct, and immune outcomes. | Infarct/immune response and recovery after VEGF-C/VEGFR3 modulation. | Effects were phase-dependent; VEGFR3 inhibition improved early outcomes, whereas acute VEGF-C delivery enlarged infarcts. Direction: timing modifies benefit versus harm. | Important preclinical cautionary evidence; strong for timing dependence, not biomarker validation. | mLV/VEGF-C modulation is not uniformly beneficial; timing is critical. |
| **Yang et al. [15]** | Experimental intervention evidence. | Male Sprague–Dawley rats (200–250 g); 90 rats randomized to dCLN removal + MCAO, AAV-VEGF-C + MCAO, or MCAO alone; 4 rats died before the 72-h endpoint and were excluded. Key assays: TTC/MRI n = 5/group; brain and spleen IHC n = 5/group; splenic flow cytometry n = 4/group; blood routine/ELISA n = 5/group. | MCAO; bilateral dCLN removal 7 days before stroke and AAV-VEGF-C administration 14 days before stroke; outcomes assessed at 3, 24, and 72 h. | Bilateral dCLN removal to block lymphatic drainage; lateral-ventricular AAV-VEGF-C as reverse validation; infarct, neurological, central/peripheral immune, and plasma cytokine readouts. | Central and peripheral inflammatory response after cerebral lymphatic blockade. | Blocking the cerebral lymphatic system reduced central and peripheral inflammatory responses. Direction: acute blockade reduced inflammation. | Preclinical intervention evidence; demonstrates bidirectional/timing-dependent biology. | Pre-stroke dCLN ablation and AAV manipulation limit clinical translation; dCLN removal is not mLV-specific and may alter cervical immune biology; follow-up was limited to 72 h. |
| **Bai et al. [48]** | Experimental recovery intervention evidence. | 136 male rats total (Sprague–Dawley and GFP-transgenic, 8–10 weeks). Main figure-level n: behavior/infarct/perfusion n = 4–7/group; angiogenesis/neurogenesis n = 4–5/group; meningeal lymphatic drainage n = 5/group; T-cell infiltration n = 6/group. | Subacute recovery phase. | Cranial bone transport with lymphatic, angiogenic, neurogenic, and behavioral readouts. | mLV drainage, repair markers, lesion burden, and neurological deficit. | Cranial bone transport enhanced mLV drainage and recovery-related markers; lesions and neurological deficits were reduced. Direction: enhanced drainage associated with repair/recovery. | Preclinical interventional recovery evidence; mechanism plausible but clinically complex. | Complex surgical intervention; translational feasibility uncertain; mainly mechanistic. |
| **Lee et al. [49]** | Experimental brain-CLN inflammatory mechanism. | 235 male C57BL/6JNarl mice (8–12 weeks); 6 saline-treated and 4 Visudyne-treated mice died after stroke surgery; 22 mice were excluded for failed surgery/bleeding and 17 for failed surgery with >10% weight loss or no spontaneous movement. Key assays: TTC infarct n = 3–6/group; MAP2 histology n = 5/group; spatial-memory tests n = 6–7/group; brain immune flow cytometry n = 8/group and CLN/spleen n = 4–8/group; scRNA-seq n = 4/group; CCN1 treatment/blockade assays n = 3–7/group. | Early reperfusion and later behavioral outcomes. | mLV disruption, scRNA-seq/inflammatory-cell analyses, and CCN1 blockade. | Reperfusion injury, inflammatory signaling, and spatial-memory deficits. | CCN1-mediated brain-CLN inflammation worsened injury; CCN1 blockade reduced infarct size and improved spatial memory.Direction: CCN1 inflammatory axis contributed to worse reperfusion injury. | Mechanistic preclinical evidence; not an imaging readout. | Not an imaging biomarker study; inflammatory mechanism may be context and timing dependent. |
| **Umehana et al. [89]** | Human exploratory indirect mLV imaging. | 11 patients with recent cerebral infarction; 6 patients with moyamoya disease as comparators. | Subacute cerebral infarction; MRI performed 11.5 ± 4.5 days after onset; delayed imaging 1–6 h after GBCA administration. | Whole-brain DANTE T1-SPACE vessel-wall MRI acquired before, immediately after, and 1–6 h after GBCA administration. | Ipsilateral delayed meningeal enhancement after infarction. | All 5 large infarctions showed ipsilateral delayed meningeal enhancement; 0/6 small infarctions did. Direction: large infarction associated with delayed ipsilateral enhancement. | Human exploratory indirect imaging; early clinical evidence but not direct flow measurement. | Very small sample; delayed enhancement is confounded by infarct size, BBB disruption, vascular enhancement, and contrast timing. |
| **Intracerebral hemorrhage - meningeal lymphatic evidence** | | | | | | | | |
| **Tsai et al. [50]** | Experimental ICH causal drainage evidence. | 294 8–12-week-old male C57BL/6 mice total. Deaths/exclusions: 2 blood-ICH and 12 collagenase-ICH deaths, 13 deaths after Visudyne ablation, 14 excluded from final analysis. Key n: mLV morphology sham day 28 n = 8, ICH day 3 n = 6, day 7 n = 8, day 14 n = 6, day 28 n = 6, day 60 n = 6; drainage sham n = 5, day 3 n = 3, day 10 n = 5, day 28 n = 6; mLV ablation hematoma n = 5/group; dCLN ligation n = 8/9 and VEGF-C n = 9/7; cilostazol behavior CMC n = 23 and cilostazol n = 22. | Acute to chronic post-ICH phases (days 3–60). | mLV manipulation; drainage assays; hematoma, iron, histology, and behavior. | Hematoma/iron clearance, neuronal/glial injury, and behavioral recovery. | Enhancing mLV function accelerated hematoma/iron clearance and recovery; ablation/blockade worsened clearance. Direction: better mLV function improved blood-product clearance and recovery. | Preclinical causal/interventional evidence; direct biological relevance to blood-product clearance. | Animal-only; dedicated human MRI biomarkers for ICH-related mLV dysfunction remain sparse. |
| **Subarachnoid hemorrhage - meningeal lymphatic evidence** | | | | | | | | |
| **Chen et al. [51]** | Experimental direct erythrocyte-clearance evidence. | Mouse SAH model; no single total n stated. RBC drainage to dCLNs: control and saline n = 12 mice/group and SAH n = 11 mice; RBC accumulation in meningeal lymphatics: control/saline n = 13 and SAH n = 15. CFSE-RBC tracing: meningeal lymphatics n = 12/group; dCLN saline n = 12 and CFSE-RBC n = 15 nodes; mandibular LN saline n = 12 and CFSE-RBC n = 23 nodes. Ablation/VEGFR3/behavior experiments used figure-level n ranging from 5/group to 22/group. | Post-SAH erythrocyte clearance phase. | Tracking extravasated erythrocytes from CSF/subarachnoid space to mLVs and dCLNs. | Erythrocyte/blood-product clearance through meningeal lymphatic routes and injury response. | mLVs cleared SAH-derived erythrocytes into dCLNs; impaired drainage aggravated SAH injury. Direction: mLV drainage contributes to blood-product clearance. | Direct preclinical biological evidence; strong mechanistic relevance for SAH blood clearance. | Mouse model; focuses on erythrocyte drainage rather than clinical MRI. |
| **Pu et al. [37]** | Experimental biological support; non-MRI. | Three-month-old male C57BL/6 mice; sham and SAH groups; tracer/histology/T-cell assays commonly n = 4/group; western blot/cytokine/apoptosis assays n = 4–5/group; behavioral assays n = 8/group. | Mainly 1 day and 1 week after SAH. | Fluorescent tracer distribution, histology, AQP4, inflammation, and behavior. | Glymphatic influx, mLV drainage to dCLNs, inflammation/apoptosis, and behavior. | SAH caused persistent glymphatic and mLV drainage malfunction. Direction: prolonged clearance dysfunction after SAH. | Preclinical mechanism; supports combined glymphatic-mLV dysfunction but not MRI validation. | Non-MRI animal study; supports mechanism but not biomarker readiness. |
| **Wang X et al. [52]** | Experimental molecular/spatial mechanism. | Male C57BL/6 mice (6–8 weeks). Main scRNA-seq used sham, SAH at 24 h, and SAH at 72 h groups, n = 10 mice/group (30 mice total), with 25,536 retained single-cell transcriptomes after QC (9,891 Sham, 8,262 SAH 24 h, 7,383 SAH 72 h). ST-seq/10x Visium was performed on dura slides; dataset reuse reports ST-seq n = 1 mouse/group. Assay-specific validation n: mLEC flow cytometry n = 4/group; dCLN fluorescent-bead drainage n = 8/group; western blot n = 3/group; behavioral tests n = 10–12/group; human CSF/prognosis association n = 48. No single total animal N was stated for all experiments. | Post-SAH meningeal injury and repair phases. | Single-cell RNA sequencing, spatial transcriptomics, and endothelial injury/apoptosis assays. | Meningeal lymphatic endothelial injury and molecular repair/apoptosis pathways. | SAH injured mLV endothelium; THBS1-CD47-related apoptotic signaling was implicated. Direction: SAH altered mLV cellular/molecular state toward dysfunction. | Mechanistic omics evidence; useful for molecular linkage, not imaging readout. | Predominantly preclinical omics; the human CSF/prognosis association was exploratory; no direct imaging biomarker or independent clinical validation. |
| **Zhu B et al. [53]** | Experimental molecular/time-course mechanism. | 117 young male C57BL/6 mice (8–10 weeks). Bioinformatics: scRNA-seq: sham, SAH at 24 h, and SAH at 72 h, n = 10 mice/group; ST-seq n = 1 mouse/group. Experiment 1: 23 mice used, 3 died; SAH at 24 and 72 h, n = 10/group. Experiment 2: 29 mice used, 3 died; mLV-hotspot IF n = 1/group, PGF/VEGF-C IF n = 4/group, PGF-blocking IF n = 4/group. Experiment 3: 65 mice used, 5 died; PGF/VEGF-C treatment and PGF-blocking behavioral groups n = 10/group. | Acute inflammation to subacute remodeling after SAH. | Spatiotemporal profiling of meningeal microenvironment and mLV function. | Meningeal inflammatory/remodeling state and mLV drainage/repair signals. | Meningeal environment shifted from acute inflammation to remodeling; PGF signaling may support early mLV repair. Direction: time-dependent remodeling influences drainage recovery. | Preclinical time-course mechanistic evidence; biologically informative but not clinical imaging. | Clinical imaging counterpart not established; assay-specific n and intervention timing limit translation. |
| **Wang J et al. [36]** | Experimental large-animal direct tracer-based MRI. | 14 male beagles: sham n = 3, SAH day 1 n = 3, SAH week 2 n = 4, and SAH + CSF drain n = 4; some MRI quantifications used n = 3 or 6. | Beagle SAH; MRI before SAH and at 1 h, 1 week, and 2 weeks. | Cisterna magna Gd-DTPA with serial T1-weighted MRI; skull-base/perineural drainage routes, cervical lymph node signal, and intermittent CSF drainage. | Meningeal lymphatic outflow along olfactory, optic, facial, vestibulocochlear, and spinal routes; CSF drainage response. | SAH caused severe early mLV outflow failure with partial recovery by 2 weeks; CSF drainage improved readouts. Direction: early delayed/reduced outflow with partial recovery. | Direct large-animal MRI evidence; strong anatomical readout but preclinical. | Small groups; invasive intrathecal contrast; anesthesia and species differences affect translation. |
| **Cai Y et al. [54]** | Experimental mechanistic/cognitive-outcome evidence. | Adult male C57BL/6J mice (8–12 weeks); 91 mice used, with 5 deaths and 6 exclusions. Key assays: Morris water maze n = 12/group; hippocampal CA1 firing n = 29 sham/27 SAH (amplitude n = 12/group, 2–3 sections/mouse); mLV morphology/junction assays n = 4–5/group; CSF drainage n = 4/group; ISF/tracer drainage n = 6/group; RNA-seq/KEGG n = 3/group; VEGF-C/VEGFR3, amyloid-β, and PI3K-AKT IF n = 5/group; in vitro LEC assays n = 3–6/group. | Cognitive and mLV outcomes assessed at 1 and 2 months after SAH. | mLV structure/drainage assays, metabolites, hippocampal function, VEGF-C treatment, and VEGFR3/PI3K-AKT signaling analyses. | mLV integrity/drainage, metabolite accumulation, hippocampal dysfunction, and spatial learning/memory. | mLV fragmentation/atrophy was accompanied by impaired drainage and cognition; VEGF-C reduced hippocampal amyloid-β deposition and improved spatial learning and memory. Direction: mLV dysfunction was associated with worse cognitive outcome, whereas VEGF-C was protective. | Preclinical mechanism linked to cognitive outcome; not clinical imaging. | Mouse model; pathway mediation remains inferential, and intervention timing and clinical translation remain uncertain. |
| **Lu et al. [90]** | Human exploratory multimodal MRI surrogate. | 58 patients with acute spontaneous SAH, an independent cohort of 26 patients with chronic post-SAH hydrocephalus, and 41 HCs. Glymphatic metrics were available for 57 patients with SAH and 41 HCs; mLV–dCLN signal-intensity measurements were available for 58 and 41, respectively; chronic-cohort analyses included 22–24 patients depending on sequence availability. | Acute spontaneous SAH and an independent cohort with chronic post-SAH hydrocephalus. | DTI-ALPS index, FW-WM, PVSVF-WM, and signal intensity of mLVs and dCLNs. | mLV/dCLN signal loss and chronic shunt-dependent hydrocephalus risk. | Compared with HCs, acute SAH was associated with a lower DTI-ALPS index, higher FW-WM and PVSVF-WM, and lower mLV and dCLN signal intensity; abnormalities were more pronounced in chronic hydrocephalus. A lower DTI-ALPS index and lower mLV signal intensity independently predicted chronic shunt-dependent hydrocephalus. Direction: lower drainage/glymphatic surrogates were associated with worse outcomes. | Human multimodal surrogate evidence; early clinical relevance but indirect. | Single-center exploratory cohort; mLV/dCLN signal intensities are indirect drainage surrogates, not direct lymphatic-flow measurements. |

Notes: AAV, adeno-associated virus; AAV9, adeno-associated virus serotype 9; ALPS, analysis along the perivascular space; AQP4, aquaporin-4; BBB, blood–brain barrier; CA1, cornu ammonis 1; CCN1, cellular communication network factor 1; CD45, cluster of differentiation 45; CD47, cluster of differentiation 47; CFSE, 5-(and 6)-carboxyfluorescein diacetate succinimidyl ester; CLN, cervical lymph node; CMC, carboxymethyl cellulose; CP, cribriform plate; CSF, cerebrospinal fluid; DANTE, delay alternating with nutation for tailored excitation; dCLN, deep cervical lymph node; DCE-MRI, dynamic contrast-enhanced magnetic resonance imaging; DTI-ALPS, diffusion tensor image analysis along the perivascular space; ELISA, enzyme-linked immunosorbent assay; FACS, fluorescence-activated cell sorting; FW-WM, free water in white matter; GBCA, gadolinium-based contrast agent; Gd, gadolinium; Gd-DTPA, gadolinium–diethylenetriaminepentaacetic acid (gadopentetate dimeglumine); GFP, green fluorescent protein; HCs, healthy controls; ICH, intracerebral hemorrhage; IF, immunofluorescence; IHC, immunohistochemistry; ILN, inguinal lymph node; ISF, interstitial fluid; KEGG, Kyoto Encyclopedia of Genes and Genomes; LEC, lymphatic endothelial cell; LN, lymph node; MAP2, microtubule-associated protein 2; MAZ51, VEGFR3 tyrosine kinase inhibitor; MCAO, middle cerebral artery occlusion; mLEC, meningeal lymphatic endothelial cell; mLV, meningeal lymphatic vessel; MRI, magnetic resonance imaging; mut, mutant; PGF, placental growth factor; PI3K-AKT, phosphoinositide 3-kinase–protein kinase B pathway; Prox1, prospero homeobox 1; PT, photothrombosis; PVSVF-WM, white matter perivascular space volume fraction; QC, quality control; qPCR, quantitative polymerase chain reaction; RBC, red blood cell; RNA-seq, ribonucleic acid sequencing; SAH, subarachnoid hemorrhage; scRNA-seq, single-cell ribonucleic acid sequencing; snRNA-seq, single-nucleus ribonucleic acid sequencing; ST-seq, spatial transcriptome sequencing; T1-SPACE, T1-weighted sampling perfection with application-optimized contrasts using different flip-angle evolutions; tdTomato, tandem dimer Tomato fluorescent protein; THBS1, thrombospondin 1; tMCAO, transient middle cerebral artery occlusion; TTC, 2,3,5-triphenyltetrazolium chloride; VEGF-C, vascular endothelial growth factor C; VEGF-C156S, VEGFR3-specific Cys156Ser variant of VEGF-C; VEGFR3, vascular endothelial growth factor receptor 3; wt, wild type; YFP, yellow fluorescent protein.The qualitative evidence/readiness column is not a formal risk-of-bias score; it summarizes biological directness and clinical translational readiness.
